# Barriers and facilitators of facility-based kangaroo mother care in sub-Saharan Africa: a systematic review

**DOI:** 10.1186/s12884-021-03646-3

**Published:** 2021-03-04

**Authors:** Mai-Lei Woo Kinshella, Tamanda Hiwa, Kelly Pickerill, Marianne Vidler, Queen Dube, David Goldfarb, Alinane Linda Nyondo-Mipando, Kondwani Kawaza

**Affiliations:** 1 Department of Obstetrics and Gynaecology, BC Children’s and Women’s Hospital and University of British Columbia, Vancouver, Canada; 2 Department of Pediatrics and Child Health, College of Medicine, University of Malawi, Blantyre, Malawi; 3 Queen Elizabeth Central Hospital, Pediatrics, Blantyre, Malawi; 4 Department of Pathology and Laboratory Medicine, BC Children’s and Women’s Hospital and University of British Columbia, Vancouver, Canada; 5 School of Public Health and Family Medicine, Department of Health Systems and Policy, College of Medicine, University of Malawi, Blantyre, Malawi

**Keywords:** Sub-Saharan Africa, Implementation, Barriers and facilitators, Kangaroo mother care, Systematic review

## Abstract

**Background:**

Hospital-based kangaroo mother care can help reduce preventable newborn deaths and has been recommended by the World Health Organization in the care of low birthweight babies weighing 2000 g or less. However, implementation has been limited. The objective of this review is to understand the barriers and facilitators of kangaroo mother care implementation in health facilities in sub-Saharan Africa, where there are the highest rates of neonatal mortality in the world.

**Methods:**

A systematic search was performed on MEDLINE, Web of Science, Cumulative Index to Nursing and Allied Health, African Journals Online, African Index Medicus as well as the references of relevant articles. Inclusion criteria included primary research, facility-based kangaroo mother care in sub-Saharan Africa. Studies were assessed by the Critical Appraisal Skills Programme Qualitative Checklist and the National Institutes of Health quality assessment tools and underwent narrative synthesis.

**Results:**

Thirty studies were included in the review. This review examined barriers and facilitators to kangaroo mother care practice at health systems level, health worker experiences and perspectives of mothers and their families. Strong local leadership was essential to overcome barriers of inadequate space, limited budget for supplies, inadequate staffing, lack of guidelines and policies and insufficient supportive supervision. Workload burdens, knowledge gaps and staff attitudes were highlighted as challenges at health workers’ level, which could be supported by sharing of best practices and success stories. Support for mothers and their families was also identified as a gap.

**Conclusion:**

Building momentum for kangaroo mother care in health facilities in sub-Saharan Africa continues to be a challenge. Strengthening health systems and communication, prioritizing preterm infant care in public health strategies and supporting health workers and mothers and their families as partners in care are important to scale up. This will support sustainable kangaroo mother care implementation as well as strengthen quality of newborn care overall. PROSPERO registration: CRD42020166742.

**Supplementary Information:**

The online version contains supplementary material available at 10.1186/s12884-021-03646-3.

## Background

Globally in 2018, 2.5 million newborns died within the first month of life, equating to approximately 7000 neonatal deaths per day [1]. Preterm birth complications is a leading cause of neonatal mortality, associated with over one in every three (36%) deaths [2]. Hospital-based kangaroo mother care (KMC), including early and continuous skin-to-skin contact of the neonate with the mother’s chest for thermoregulation and bonding, breastfeeding support and early discharge from hospital, has the potential to halve the number of preterm deaths [3]. Although KMC is recommended by the World Health Organization (WHO) for babies weighing 2000 g or less, implementation has been limited [4]. This highlights the need to understand barriers and facilitators to sustainable implementation of KMC, which is especially important in sub-Saharan Africa (sSA) where neonatal mortality rates are the highest in the world (28 per 1000 live births as compared to a global rate of 18 per 1000 live births) [1]. The objective of this review is to understand barriers and facilitators of KMC in health facilities in sSA.

## Methods

Searches were conducted on MEDLINE Ovid, Web of Science, Cumulative Index to Nursing and Allied Health (CINAHL), African Journals Online (AJOL) and the WHO Regional Database for Africa, African Index Medicus (AIM) from database inception to December 2019, with no limits applied to the year of publication or language. Searches were supplemented by scanning reference lists of papers included for review. Based on the PICOS research framework (Table 1), search included MESH terms Kangaroo-Mother Care Method and “Africa South of the Sahara” and keywords broadly included kangaroo mother care, skin-to-skin care and countries in sub-Saharan Africa (Table 2 for detail on search terms). Results were manually screened for implementation factors, barriers and facilitators and facility-based care to prevent missing relevant studies in the original search that did not include these keywords. A review protocol detailing the research question, search strategy, inclusion and exclusion criteria, quality assessment and strategy for data synthesis was developed in consultation with pediatric clinicians from Malawi (TH, QD, KK) to refine the scope of the review and ensure relevance to sSA contexts. The protocol was registered to Prospero (CRD42020166742).

**Table 1:** PICOS research framework

**P**opulation	Mothers and newborns dyads, health facilities and health workers practicing facility-based KMC
**I**ntervention	Facility-based KMC
**C**ontext	Health facilities in sSA with inpatient KMC
**C**omparisons	Conventional methods of care, incubator care, N/A
**O**utcome	Barriers and facilitators of facility-based KMC practice
**S**tudy	Experimental studies (controlled trials) and observational studies (cohort, case-controlled, cross-sectional, qualitative)

**Table 2:** Search terms

Intervention	Kangaroo-Mother Care Method/ “kangaroo mother care” OR “kangaroo care” OR “KMC” OR “skin to skin” OR “skin-to-skin” OR “STS care”
Context	“Africa South of the Sahara”/ Africa or sub-Sahar* or south* Africa or west* Africa or east* Africa or Angola or Benin or Botswana or Burkina Faso or Burundi or Cameroon or Cameroons or Cameron or Camerons or Cape Verde or Cabo Verde or Central African Republic or Chad or Comoros or Comoro Islands or Comores or Mayotte or Congo or Zaire or Cote d’Ivoire or Ivory Coast or Djibouti or French Somaliland or Eritrea or Ethiopia or Gabon or Gabonese Republic or Gambia or Ghana or Guinea or Guinea-Bissau or Kenya or Lesotho or Basutoland or Liberia or Madagascar or Malawi or Nyasaland or Mali or Mauritania or Mauritius or Mozambique or Namibia or Niger or Nigeria or Rwanda or Ruanda or “Sao Tome and Principe” or Senegal or Seychelles or Sierra Leone or Somalia or South Africa or Sudan or South Sudan or Swaziland or Eswatini or Tanzania or Togo or Uganda or Zambia or Zimbabwe or Rhodesia

Two reviewers (MWK, TH) independently screened titles and abstracts according to the eligibility criteria (Table 3). Discrepancies were resolved by discussion and a third reviewer (KP) was asked to adjudicate in the absence of consensus. Full texts were then independently reviewed by the two reviewers (MWK, TH) with the third reviewer (KP) providing an independent assessment in any disputes regarding eligibility. Studies were screened for inclusion if they included mothers and newborns dyads who practiced facility-based KMC in sSA countries as well as if they included health facilities and health workers that implemented KMC in their institutions. Studies that did not specify KMC as a therapeutic practice separate from routine skin-to-skin contact were excluded. Additionally, we excluded studies that explored community-based KMC, such as home visits, outpatient care, and interviews with community health workers or village members. Since our research objective focused on facility-based KMC, studies without primary data collection in health facilities by study authors were excluded, such as review articles and protocols. We evaluated quantitative studies using the study quality assessment tools of the National Heart, Lung, and Blood Institute of the National Institutes of Health (NIH) [5] and qualitative studies using the Critical Appraisal Skills Programme (CASP) Qualitative Checklist [6]. An overall study rating based on critical concerns of internal validity was added to the CASP checklist to consider quality assessment similar to the NIH quality assessment tools. Results reported according to the Preferred Reporting Items for Systematic Reviews and Meta-Analyses guidelines (PRISMA) [7].

**Table 3:** Eligibility criteria

Inclusion criteria	Exclusion criteria
Studies conducted in sSA countries	Studies not conducted in sSA countries
KMC in health facilities	Community based KMC, home visits by health care workers
Published experimental and observational studies including randomized or non-randomized trials, cohort, case-controlled, cross-sectional survey, facility evaluations and qualitative studies	Studies without primary data collection, such as reviews and study protocols, as well as those not demonstrating clear research methodology, including abstracts, conference proceedings, commentaries, letters and editorials.
Mother infant dyads including premature, low birthweight (LBW) (as defined by individual study authors) and term neonates (≤ 28 days)	Studies with older infants, studies without human subjects

Details about study country, facility type, rural or urban context, study design, sample size, newborn characteristics, KMC characteristics, onset of skin-to-skin care, barriers and facilitators were extracted into Excel (Microsoft, Redmond, United States). Two reviewers (MWK, KP) independently extracted data and conducted the quality assessment from a sample of eligible studies (three studies, 10%) until agreement was achieved, with the remainder extracted by one reviewer (MWK). The data extraction sheet was imported into NVivo 12 (QSR International, Melbourne, Australia) where thematic analysis was conducted of barriers and facilitators according to health system and facility, health worker and family level factors. Excerpts that did not fit into the specified categories were coded as ‘other’ and reviewed for emergent themes. Illustrative quotes and excerpts from the studies were extracted to highlight key themes.

## Results

**Fig. 1:**
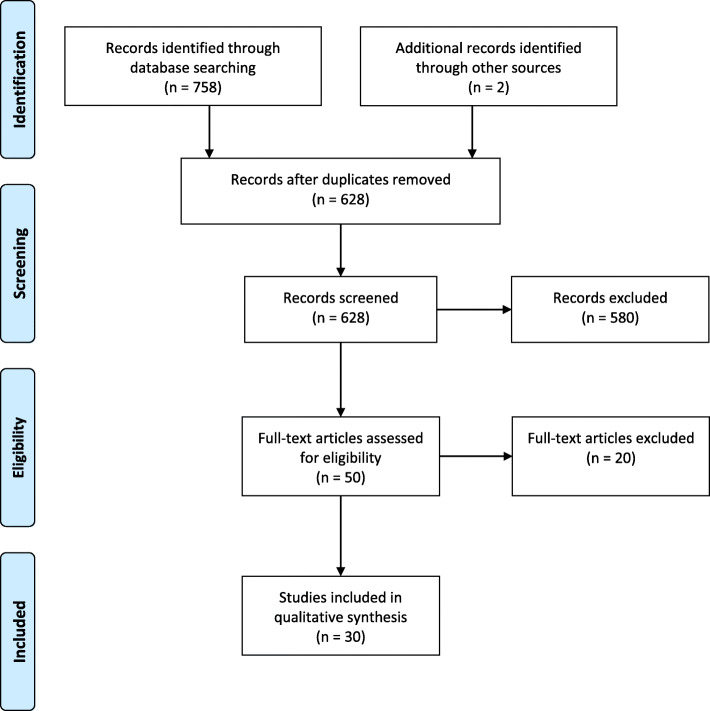
Prisma flow diagram

We identified a total of 761 publications from our database searches (199 from Medline, 151 from Web of Science, 90 from CINAHL, 318 from AIM and 1 from AJOL) and reference lists (2 references). After removal of duplicates and screening against the eligibility criteria, 30 studies were included in the review (Fig. 1). Reasons for exclusion included lack of focus on reporting barriers or facilitators to KMC implementation (*n* = 12), KMC in community settings rather than in health facilities (*n* = 3), review or conference proceedings (*n* = 3), discussed development of evaluation tools but did not report results (*n* = 1) and not in English (*n* = 1).

The number of publications discussing barriers and facilitators of implementation of facility-based KMC in sSA has steadily grown over the past two decades from two prior to 2000, nine between 2000 and 2009 and 19 from 2010 to 2019 (Additional file 1 for characteristics of included studies). There were two multi-site studies: one based in Malawi, Mali, Rwanda and Uganda [8], while the other was conducted in Ethiopia, Indonesia and Mexico [9]. For this review focusing on sSA, only the Ethiopian results were included. Of the studies in single sSA countries, five were from West Africa (three from Ghana, two from Nigeria), 17 from Southern Africa (three from Malawi, two from Mozambique, nine from South Africa, one from Zambia, two from Zimbabwe) and six from East Africa (one from Ethiopia, one from Tanzania, four from Uganda). Twenty-one studies, a majority of those included in the review, were conducted in tertiary and secondary level hospitals (70%), while six studies included both hospitals and health centres (21%). Facility level was not clear in three (10%). Tertiary-level hospitals included central, university teaching hospitals and hospitals with specialized maternity and neonatal units while secondary-level hospitals included regional referral, district and rural hospitals. A majority of studies were conducted in urban areas, including 17 (57%) in urban health facilities, 8 (27%) in urban and rural facilities and only one study (3%) in rural settings alone. There were four studies with unclear settings.

Newborn characteristics were not described in ten studies (33%). Of those with newborn data, nine described prematurity and LBW (30%) and seven described LBW alone (23%) as eligibility for KMC. The least frequently described indicator was prematurity alone, which was found in four studies (13%). Cut-offs for LBW varied between below 2500 g to 1800 g for KMC initiation. Five studies (17%) described the use of KMC for stable newborns only, while two studies (7%) included clinically unstable ones. KMC components were not detailed in 13 studies (43%). For nine studies (30%), KMC was synonymous with skin-to-skin care. KMC involved skin-to-skin care and exclusive breastfeeding in three studies (10%). Another three studies (10%) described KMC as skin-to-skin care, exclusive breastfeeding and early discharge, while two studies (7%) added a fourth component of maternal support. Twenty studies did not provide details regarding the onset of skin-to-skin care (67%), though five studies noted KMC initiation immediately after birth with postnatal mothers admitted to KMC units with premature or low birthweight infants (LBWI) (17%) and five studies described initiation of KMC once the newborn was eligible (17%). In the latter, eligible newborns may be identified during ward rounds in the neonatal unit according to clinical stability requirements.

Overall, the majority of studies were considered of good or fair quality, indicating internal validity of reported results (Additional file 2). Those rated poorly were frequently due to limited reporting of methods, which led to unclear assessments of quality. Ten of the 13 qualitative studies were rated good or fair. Those rated poor did not clearly describe their methods of qualitative analysis [10–13]. The single case series study was rated fair; although the objective, study population, intervention and outcome were clearly described, there was lack of clarity on case characteristics [14]. Seven of the 11 observational cohort and cross-sectional studies were rated good or fair. Among poor studies, lack of clarity existed on refusal rate of eligible participants and ambiguity on methods of data collection and outcome indicators [15–18]. Among the four controlled intervention studies, two were rated fair. Potential sampling bias, presence of confounders and lack of reporting compliance to treatment increased the risk of bias in the two poor studies [9, 19].

### Health system and facility factors

**Fig. 2:**
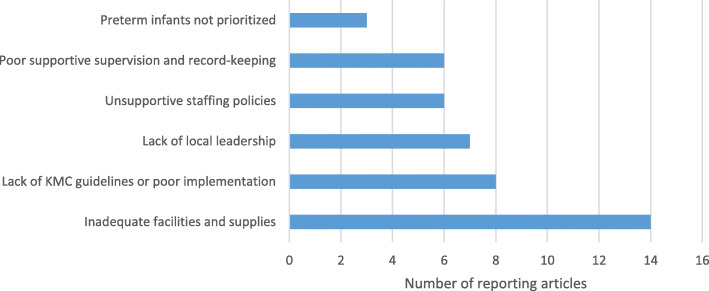
Health system barriers for KMC

Twenty-one studies (70%) described health system and facility-based barriers and 20 (67%) described facilitators of KMC initiation. Inadequate facilities and supplies were most frequently described as barriers to implementation (Fig. 2). These include lack of dedicated space for KMC, not enough beds, shortage of chairs for mothers, lack of privacy and issues of overcrowding-- as well as not having hats for newborns and cloth wrappers to facilitate KMC or equipment like functional weighing scales or monitoring devices [9, 11, 14, 15, 17, 18, 20–26]. A study in Uganda found that intermittent skin-to-skin care was practiced instead of continuous care due to lack of suitable environments in 75% of cases, including lack of beds and space for relatives supporting mothers in the KMC area [14]. Another frequently mentioned barrier was lack or poor implementation of KMC guidelines, including policies, protocols and job aids [8, 10, 17, 18, 24–27]. A study in Malawi, for example, found that while policies were implemented at national level, 63% of health workers in health centres and district hospitals did not know of their existence [26]. Inconsistent local leadership was also a frequently reported barrier [8, 11, 22, 23, 25, 27–29]. A Ugandan study found that training, supportive supervision and resources were provided by external partners: *“So far I’ve only seen Save [the Children]”* [27]. A health worker managing a neonatal unit in South Africa reflected, *“I am no longer in charge of the unit … Unfortunately, my old unit is now leaderless as they have not appointed anyone in my place … and no one is really promoting or teaching KMC”* [11]. Unsupportive staffing policies such as rotations and allocations, which compromised ability to retain trained staff in neonatal units and orientate new staff [22, 24–27, 30], poor supportive supervision and record-keeping [8, 13, 24–27] and low priority given to preterm infants [8, 23, 26] were also described as barriers. Two studies highlighted that KMC may be less utilized in private health facilities, perhaps due to concerns about costs for extended hospitalization [17, 31].

**Fig. 3:**
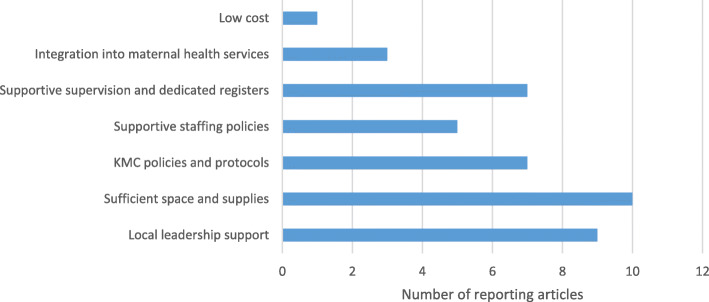
Health system facilitators for KMC

The most frequently described facilitator was sufficient space and supplies [9–11, 18, 20, 31–34] followed by local leadership support [16, 18, 22–24, 27, 29, 32, 35] (Fig. 3). A dedicated space for KMC with enough beds, bed linens and cloth wrappers helped to facilitate KMC. A study in Ethiopia found that health facilities with separate newborn areas were 49% more likely to initiate KMC than those without (aOR 1.49; 95% CI 1.06–2.10) [31]. Further restructuring KMC space to include a dayroom with TV, dining area, laundry area, lockers and cupboards, bed linens, reclining beds and chairs and meals supported longer hospital stays. Involvement of senior management such as hospital directors, medical superintendents, head nurses and nursing managers helped to build a culture of KMC practice and allocate resources. A study in Uganda highlighted how presence of the hospital director in all meetings strengthened efforts and support of senior management allowed for staff to be trained and space alterations [27]. Written KMC policies that clearly outlined roles, responsibilities and procedures [10, 11, 17, 24, 26, 29, 32] as well as supportive supervision and improved accountability through dedicated KMC registers [11, 16, 24, 25, 27, 29, 32] were also frequently mentioned. Other facilitators included supportive staffing policies such as not rotating staff [10, 11, 16, 24, 31], integrating KMC into maternal health care such as during antenatal counselling [14, 24, 32] and lower costs in comparison to incubators that have high demands for electricity [9].

### Health worker factors

**Fig. 4:**
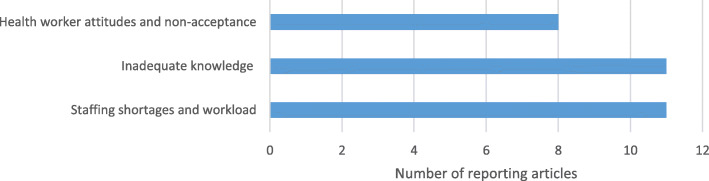
Health worker barriers for KMC

On health worker level, 17 studies (57%) described barriers while 16 studies (53%) described facilitators. Staffing shortages and workload were the most frequently reported barriers for health workers to effectively implement KMC [10, 11, 15, 16, 20, 22, 23, 26, 28, 31, 33] (Fig. 4). Nurses in a Mozambican study shared that *“they had no time to check temperature, to weigh infants, to supervise breastfeeding and to talk to mothers, especially in the afternoon and night shifts when two nurses had to deal with 60-80 patients”* [16]. Inadequate knowledge of KMC and its benefits was also frequently reported as a barrier, which was associated with health workers’ lack of confidence [10, 11, 15–18, 20, 23, 24, 27, 34]. A study in Ghana found that over a third of nurses (36%) did not talk to mothers about KMC because they lacked adequate knowledge to counsel [10]. Additionally, KMC was largely perceived as skin-to-skin care for thermoregulation, while bonding and exclusive breastfeeding in 52% and early discharge in 69% were not known as components of KMC [10]. Health workers’ attitudes and non-acceptance were also highlighted as barriers to practice [11, 13, 15–17, 23, 26, 28]. Nurses may be skeptical of KMC and believe that *“only better equipment and supplies would improve survival of LBWI”* as found in a Mozambican study [16] or an underlying belief that preterm infants were unlikely to survive, so less support was given to KMC mothers and infants as found in a Malawian study [26].

**Fig. 5:**
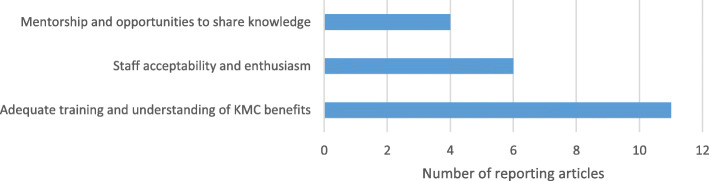
Health worker facilitators for KMC

The most frequently mentioned facilitator to supporting health workers’ practice of KMC was adequate training (Fig. 5). This training should include the different components and benefits of KMC. Effective training methods were pre-service curricula in nursing and medical programs, complemented by continuous in-service training, face-to-face facilitation with multimedia materials and training sessions, coordinated by regional levels and refreshed by meetings, workshops and exposure to current literature on the topic [8, 10, 11, 15, 16, 20, 26, 29, 33, 35]. An initially resistant service provider from South Africa shared that she became an enthusiastic advocate when *“Finally, I understood the objectives – decreased infection, more successful breastfeeding, improved homeostasis and decreased hospital stays. These were things for which I could advocate. It made sense”* [11]. Additionally, training can help dispel misconceptions: in Ghana almost all nurses in that study (66 of 67; 93%) knew that HIV positive mothers could safely provide KMC [10]. Staff acceptability and enthusiasm for KMC were also reported as facilitators to sustainable practice [9–11, 16, 23, 28, 29] and nurses from Mozambique reported feeling proud to be able to successfully manage LBWI in their facilities, indicating increased referral rates as a sign of recognition of their new skills [16]. Mentorship and opportunities to share knowledge were also highlighted as ongoing methods of engaging staff to support KMC practice including development of health workers KMC ‘champions’ to support scale-up [34], peer-led workshops and mentorship visits [32], periodic discussion of results between doctors and nurses [16] and sharing knowledge through professional and hospital networks [29].

### Mothers and families

**Fig. 6:**
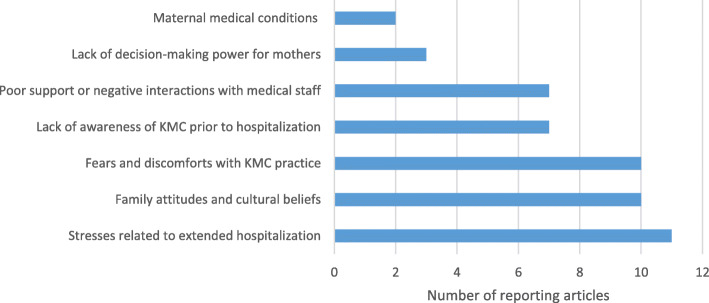
Family-level barriers for KMC

Barriers to practice KMC were described in 22 studies (73%) and facilitators in 20 studies (67%) for mothers of premature newborns. The most frequently mentioned barrier to sustain KMC practice was stress related to extended hospitalization including additional costs to families to support living expenses of mothers and infants in the hospital and concerns about responsibilities at home such as care of other children [9, 10, 12, 17, 18, 20, 24, 26, 30, 33, 34] (Fig. 6). Hospitals reported not supplying adequate food, urging family members to bring food or money [12, 27, 33, 34]. Families’ attitudes and cultural beliefs that babies should be carried on the back not on the front, guilt related to having premature infants and lack of motivation due to skepticism that these could survive, were also frequently mentioned [8, 10, 11, 16, 19, 23, 26, 27, 30, 33]. Fears and discomforts with KMC were frequently reported [10, 12, 15, 18, 19, 21, 30, 33, 36, 37]. Some mothers reported anxieties around handling their small babies. Backache from continuous positioning the infant on the chest, difficulties with sleeping and tiredness, boredom and isolation as the mother is separated from her family, were also shared during practicing KMC [37]. Other barriers included lack of awareness of KMC prior to birth [13, 14, 18, 21, 30, 34, 37], poor support or negative interactions with medical staff [12, 13, 20, 21, 24, 30, 33], decision-making heavily influenced by grandmothers and fathers not engaged in KMC counselling [12, 30, 37] and maternal medical conditions such as caesarean births that hinder skin-to-skin contact [14, 33].

**Fig. 7:**
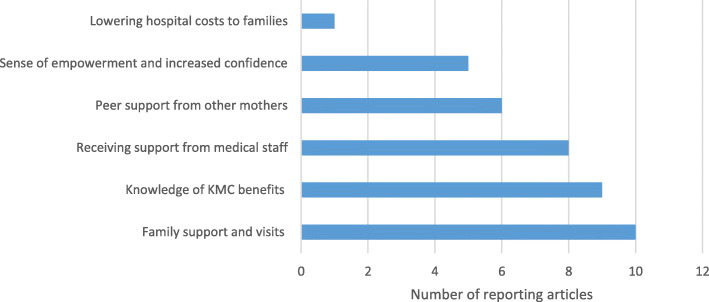
Family-level facilitators for KMC

Overall, support from family [8–10, 12, 14, 20, 23, 29, 33, 36], peers [14, 18, 21, 36, 37] and health workers [12, 16, 18, 32, 33, 36] were important in facilitating KMC practice (Fig. 7). Family support included frequent hospital visits to bring money and supplies, companions who stayed with the mother to support care and approval of fathers and grandmothers, being influential decision-makers, helped to alleviate worries of home. Peer support included support from other mothers in the ward on advice for positioning and emotional and practical support from health workers, including KMC positioning, continual reassurance that infants are doing well and talking about their fears, was also mentioned. Knowledge of KMC benefits [9, 12, 14, 18–20, 24, 30, 36] and a sense of empowerment [12, 32, 33, 36, 37] were reported as facilitators to practice. In Zambia, mothers with high knowledge were almost four-fold more likely to practice KMC (knowledge score 0–11 vs 12–15; aOR 3.88; 95% CI:1.13–13.29) [20]. In South Africa, infant weight gain led to feelings of excitement and increased determination and commitment by mothers as they gained confidence in KMC [36]. In Nigeria, the need was mentioned to lower hospital costs to families to support an extended stay during KMC [17].

Barriers and facilitators to KMC practice by study are reported in Additional files 3 and 4.

## Discussion

This review focused on facility-based KMC implementation in sSA. At health system level, strong local leadership was essential to overcome barriers of inadequate space, budget, staffing allocation, lack of guidelines and supportive supervision. At health worker level, workload and knowledge gaps compounded with staff skepticism of KMC were barriers to KMC practice. Health worker champions and continued engagement with health workers through mentorship visits were important to build critical mass in KMC practice. Lastly, at mother and family level, mothers required support from their families, peers and health workers while practicing KMC.

A previous review ranking barriers and facilitators from studies in low- and middle-income countries (LMICs) highlighted similar issues [38]. Low awareness of KMC, insufficient facility space, supplies and fears of harming the small infant were important barriers for mothers in LMICs, while workload, lack of clear guidelines, training and belief in its efficacy were barriers to adopt KMC for nurses [38]. Similar findings in our review suggest building momentum for sustainable KMC implementation in sSA continues to be a challenge, perhaps due to the complexity of KMC requiring strong engagement by users and stakeholders [4]. Leadership, governance and health workforce building were reported as significant bottlenecks to scaling up KMC in sSA and Asia [39]. Effective adoption of KMC practice requires synergy of effort and alignment by health policy makers, senior management in health facilities, nurses and other health workers at the front-line and mothers and families who often face challenges in effective communication and sharing of best practices.

Our review adds to the existing literature by highlighting the interaction of health system, health worker and mother levels, particularly in the cross-cutting theme of prioritizing preterm infant health. As a policy maker in Malawi noted, prematurity was not an area of focus until the 2012 ‘Born Too Soon’ report on preterm birth, revealing nine of the 11 countries with preterm birth rates of ≥15% were in sSA, including Malawi with the world’s highest rate of 18% [26]. At health systems level, prioritizing the health of preterm infants includes investment in facilities and policies to integrate education about prematurity into maternal health care. Staffing policies where neonatal care nurses are not rotated, may strengthen health worker specialization with small and sick newborns as some have questioned the sustainability of attempting to continually orient new staff [27]. At health worker level, prioritizing the health of preterm infants highlights the need to address staff attitudes that preterm babies are unlikely to survive. This may be supported through sharing of best practices and success stories with KMC. At mothers and family level, some studies in this review highlighted the shock of parents unprepared for preterm birth and the struggle to cope emotionally with the fear of potentially losing their infants, and cope practically with an extended stay in hospital. This could be supported by sensitization during antenatal care and birth preparedness that highlights potential for prematurity, especially in many sSA countries where rates are high and there may be stigma around preterm birth. In other words, lower prioritization at health systems level relates to less training and specialization for health staff as well as less preparation among families. This reduces capacities for sustainable implementation of KMC as well as other neonatal innovations. Similar barriers of staffing rotation policies, understaffed neonatal units and inadequate support provided to families can be seen with the implementation of bubble continuous positive airway pressure in sSA, an intervention to support care of preterm and LBWI with respiratory distress [40].

Different conceptualizations of what constitutes KMC emerged in our review. Two-thirds of studies were ambiguous on the onset of KMC practice and supporting breastfeeding appeared to be a potentially neglected area. Previous reviews described exclusive breastfeeding as a component of KMC and mentioned that breastfeeding challenges were a barrier to continued KMC practice [4, 38]. Thirty percent of studies in our review only described skin-to-skin care in KMC practice. In Ghana, most health workers agreed that skin-to-skin contact for thermoregulation and bonding was a component of KMC, but less than half included exclusive breastfeeding [10]. Consideration of issues around supporting breastfeeding is important, especially as preterm and LBWI may require expressed breastfeeding, which family members may not be familiar with [41, 42]. Family members may measure progress only in terms of weight gain [37]. Poor weight gain is unhealthy, but also negatively impacts the morale of mothers and health workers [37]. A focus on strengthening breastfeeding also highlights the importance of maternal nutrition and provision of food. Ensuring an adequate and nutritious diet in the hospital would help support health and well-being of the mother, as well as support breastfeeding, which in turn may alleviate tensions arising from dependency on family support.

Lastly, studies from sSA in this review emphasized the complexities of donor-funded KMC programs and challenges of inconsistent local leadership, where external partners propelled momentum. Funding for prenatal and neonatal health has increased substantially [43]. Global health initiatives have led to improvements in health outcomes over the past decade, although coordination with national governments and follow-up continues to be a challenge [44]. For KMC studies, donor-led implementation can lead to extra resources for scale-up, including supporting KMC champions for building momentum in practice, but quality of care may deteriorate as projects end. Continued engagement of health workers through supportive supervision, mentorship and knowledge sharing of best practices and local leadership in prioritization of prenatal and neonatal health is required in the long-term for institutionalization of KMC.

## Conclusion

Although KMC is a relatively simple intervention in concept, sustainable implementation requires the combined support of health systems, health workers, mothers and families. In sSA where a heavy burden of neonatal deaths exists, building momentum for KMC involves strengthening health systems and communication, prioritization of preterm infant health in public health strategies and supporting health workers and mothers with their families as partners in care. This is important in supporting KMC, but also in maternal and newborn care in general.

## Supplementary Information

**Additional file 1.** Characteristics of included studies.

**Additional file 2.** Quality assessment.

**Additional file 3.** Barriers to KMC practice by study.

**Additional file 4.** Facilitators of KMC practice by study.

## Data Availability

All data generated or analysed during this study are included in this published article and its supplementary information files.

## Abbreviations

AJOL: African Journals Online database
AIM: African Index Medicus database
CASP: Critical Appraisal Skills Programme
CINAHL: Cumulative Index to Nursing and Allied Health database
KMC: Kangaroo mother care
LBW/ LBWI: Low birthweight / low birthweight infants
LMIC: Low- and middle-income countries
NIH: National Institutes of Health
PRISMA: Preferred Reporting Items for Systematic Reviews and Meta-Analyses guidelines
sSA: Sub-Saharan Africa
WHO: World Health Organization
