# Supplementary material for: Barriers and facilitators of facility-based kangaroo mother care in sub-Saharan Africa: a systematic review
Source: BMC Pregnancy Childbirth. 2021 Mar 4;21:176. doi: 10.1186/s12884-021-03646-3 (PMC7934357; doi:10.1186/s12884-021-03646-3)
Supplement: Supplementary file 1 — Additional file 1. Characteristics of included studies. [file 12884_2021_3646_MOESM1_ESM.docx]

**Additional file 1: Characteristics of included studies**

| **Reference** | **Country** | **Study design** | **Facility type** | **Rural or urban** | **Sample size** | **Newborn characteristics** | **KMC components** | **Onset of skin-to-skin care** |
| --- | --- | --- | --- | --- | --- | --- | --- | --- |
| Adzitey et al 2017 | Ghana | Descriptive, interviews | Tertiary- and secondary-level hospitals | Urban | 67 nurses | Preterm and LBWI (cut offs not described) | Skin-to-skin care, exclusive breast-feeding, early discharge and maternal support | Immediately after birth (70% of respondents) |
| Aliganyira et al 2014 | Uganda | Facility evaluation, focus group/ interviews | Hospitals and health centres that provide maternal and newborn services | Mixed | 11 health facilities | N/A | Skin-to-skin care | N/A |
| Bergh and Pattinson 2003 | South Africa | Facility evaluation, interviews | Tertiary- level hospitals | Urban | 2 facilities | N/A | N/A | N/A |
| Bergh et al 2008 | South Africa | Randomized controlled trial | N/A | Mixed | 36 facilities | N/A | N/A | N/A |
| Bergh et al 2012 | Ghana | Implementation process evaluation, facility evaluation after 1 year of implemen-  tation | Tertiary- and secondary-level hospitals | Mixed | 38 facilities | N/A | N/A | N/A |
| Bergh et al 2013 | Ghana | Facility evaluation after end of project | Tertiary- and secondary-level hospitals | Mixed | 38 facilities | N/A | Skin-to-skin care and exclusive breastfeeding | Immediately after birth |
| Bergh et al 2014 | Malawi, Mali, Rwanda and Uganda | Facility evaluation, focus group/ interviews | Hospitals and health centres that provide maternal and newborn services | Mixed | 39 facilities | N/A | Skin-to-skin care | N/A |
| Cattaneo et al 1998 | Ethiopia (as well as Indonesia and Mexico) | Randomized controlled trial | Tertiary-level hospital | Urban | 100 LBWI, singleton births (in Ethiopia) | Stable LBWI (1000 to 1999g) | Skin-to-skin care and exclusive breastfeeding | Once eligible (see newborn characteristics) |
| Chavula et al 2017 | Malawi | Cross-sectional facility evaluation (Emergency Obstetric Newborn Care Survey 2014 from Malawian MoH) | Tertiary- and secondary-level hospitals | Mixed | 87 facilities | Stable preterm and LBWI (≤2000g) | Skin-to-skin care and exclusive breastfeeding | Once eligible (as soon as they are clinically stable) |
| Chisenga, Chalanda, and Ngwale 2015 | Malawi | Descriptive, focus group/ interview | Tertiary-level hospitals | Urban | 113 mothers | Preterm and LBWI (<2500g) | N/A | N/A |
| Davidge 2009 | South Africa | Descriptive, key informant reflection | Tertiary-level hospital | Urban | 1 facility | N/A | Skin-to-skin care, exclusive breastfeeding, early discharge and maternal support | N/A |
| Feucht et al 2015 | South Africa | Implementat-ion process evaluation, facility evaluation | Tertiary & secondary level hospitals | N/A | 8 facilities | N/A | N/A | N/A |
| Gondwe et al 2016 | Malawi | Descriptive, interviews | Hospitals and health centres that provide maternal and newborn services | N/A | 11 health care  providers& five policy makers | Preterm infants | N/A | N/A |
| Ibe et al 2004 | Nigeria | Controlled intervention (cross-over), interviews | Tertiary- level hospital | Urban | 13 newborns, 11 mothers and female relatives | LBWI (1200-1999g) | Skin-to-skin care | After enrollment |
| Kambara-mi, Chidede and Kowo 1999 | Zimbaba-we | Cross-sectional survey | Tertiary- level hospital | Urban | 613 mother infant pairs | Stable preterm and LBWI (<2000g) that were able to suck | Skin-to-skin care | Once eligible identified during ward rounds in the neonatal unit |
| Kambara-mi, Mutambir-wa and Maramba 2002 | Zimbaba-we | Descriptive, focus group/  interview | Tertiary- level hospital | Urban | Four focus groups with 10-12 people | Stable preterm and LBWI (cut offs not described) | N/A | N/A |
| Kampekete, Ngoma and Masumo 2018 | Zambia | Cross-sectional survey | Tertiary- level hospital | Urban | 60 mothers, 5 health workers managing KMC unit | Premature and LBWI (<2000g) | Skin-to-skin care, exclusive breastfeeding and early discharge | Immediately after birth |
| Kiwanuka et al 2017 | Tanzania | Descriptive, focus group/  interview | Secondary- level hospital | Urban | 15 mothers | Preterm and LBWI (cut offs not described) | N/A | N/A |
| Leonard and Mayers 2008 | South Africa | Descriptive, focus group/  interview | Tertiary- level hospital | Urban | 6 parents | Premature infants | N/A | N/A |
| Lincetto, Nazir and Cattaneo 2000 | Mozambique | Prospective cohort | Secondary- level hospital | Urban | 22 babies | LBWI (≤1800g) irrespective of gestational age, place of birth and health condition | Skin-to-skin care,  exclusive breast-feeding,  discharge if ≥1500g and/ or increased weight for ≥3 consecutive days | Once eligible As soon as possible after admission to all 1800g or less |
| Morgan et al 2018 | Uganda | Facility audit, interviews and feasibility study | Secondary- level hospital | Urban | 254 neonates in the audit, 10 neonates in feasibility study, 20 health workers in accep-tability study | Clinically unstable LBWI (≤2000g) | Skin-to-skin care | Once eligible, clinically  unstable  singleton liveborn infants <48 hours delivered at Jinja Hospital with birthweight ≥700 and ≤2000g and mother available |
| Namazzi et al 2015 | Uganda | Facility evaluation (nested within a randomized controlled trial of a community intervention) | Hospital and health centres that provide maternal and newborn care | Rural | 20 facilities (KMC impleme-nted at the one district hospital) | Preterm infants | Skin-to-skin care | N/A |
| Onubogu and Okoh 2016 | Nigeria | Cross-sectional survey | Hospitals and health centres that provide maternal and newborn services | N/A | 157 doc-tors and nurses partici-  pating in PaedAss-Nigeria conf2014 | Preterm and LBWI (cut offs not described) | N/A | N/A |
| Pattinson et al 2005 | South Africa | Randomized controlled trial | N/A | Mixed | 34 facilities | N/A | N/A | N/A |
| Reddy and McInerney 2007 | South Africa | Descriptive, interviews | Secondary- level hospital | Urban | 10 mothers | LBWI <2000g | Skin-to-skin care | N/A |
| Söderbäck and Erlandsso-n 2012 | Mozamb-ique | Descriptive, ethnographic observation, interviews | Tertiary-  level hospital | Urban | 41 mothers | Preterm infants | Skin-to-skin care | N/A |
| Solomons and Rosant 2012 | South Africa | Cross-sectional survey | Secondary- level hospital | Urban | 30 mothers, 6 nurses | Low LBWI <2500g | N/A | N/A |
| ten Ham, Minnie and van der Walt 2016 | South Africa | Descriptive, interviews | N/A | N/A | 13 health professionals | N/A | Skin-to-skin care and exclusive breastfeeding | N/A |
| Watkins et al 2018 | Uganda | Case series | Secondary- level hospital | Urban | 12 newborns | Stable LBWI <2000g hospital-born mother able to participate in KMC | Skin-to-skin care | Immediately after birth |
| Weldearegay et al 2019 | Ethiopia | Cross-sectional facility evaluation (Emergency Obstetric Newborn Care Survey 2016 from Ethiopian MoH) | Hospitals and health centres that provide maternal and new-born care | Mixed | 293 hospitals, 3459 health centres, 52 clinics | Preterm and LBWI (<2000g) | N/A | N/A |

*KMC - kangaroo mother care; LBWI – low birthweight infant; MoH – Ministry of Health*
