# Supplementary material for: Barriers and facilitators of facility-based kangaroo mother care in sub-Saharan Africa: a systematic review
Source: BMC Pregnancy Childbirth. 2021 Mar 4;21:176. doi: 10.1186/s12884-021-03646-3 (PMC7934357; doi:10.1186/s12884-021-03646-3)
Supplement: Supplementary file 2 — Additional file 2. Quality assessment. [file 12884_2021_3646_MOESM2_ESM.docx]

**Additional file 2: Quality assessment**

**QUALITATIVE (CASP checklist)**

*Used for descriptive studies that employed focus group discussions and/or interviews in their methods*

| **Reference** | **1** | **2** | **3** | **4** | **5** | **6** | **7** | **8** | **9** | **10** | **Rating** |
| --- | --- | --- | --- | --- | --- | --- | --- | --- | --- | --- | --- |
| Adzitey et al 2017 | Y | Y | CD | Y | Y | CD | N | N | Y | Y | Poor |
| Aliganyira et al 2014 | Y | Y | Y | Y | Y | CD | Y | N | Y | Y | Fair |
| Bergh and Pattinson 2003 | Y | Y | Y | Y | Y | Y | N | Y | CD | Y | Fair |
| Bergh et al 2014 | Y | Y | CD | CD | Y | CD | Y | Y | CD | Y | Fair |
| Chisenga et al 2015 | Y | Y | Y | Y | Y | CD | Y | Y | Y | Y | Good |
| Davidge 2009 | CD | Y | CD | CD | CD | N | N | N | Y | CD | Poor |
| Gondwe et al 2016 | Y | Y | Y | Y | Y | CD | Y | Y | Y | Y | Good |
| Kambarami et al 2002 | Y | Y | Y | N | Y | CD | N | N | Y | Y | Poor |
| Kiwanuka et al 2017 | Y | Y | Y | Y | Y | N | Y | CD | Y | Y | Fair |
| Leonard and Mayers 2008 | Y | Y | Y | Y | CD | CD | Y | CD | Y | Y | Fair |
| Morgan et al 2018 | Y | Y | Y | Y | CD | CD | Y | Y | Y | Y | Good |
| Reddy and McInerney 2007 | Y | Y | Y | Y | CD | CD | Y | Y | Y | Y | Fair |
| Söderbäck and Erlandsson 2012 | Y | Y | Y | CD | CD | CD | N | CD | Y | CD | Poor |
| ten Ham et al 2016 | Y | Y | Y | Y | Y | CD | Y | Y | Y | Y | Good |

*Y – Yes, N – No, CD – Cannot determine/ cannot tell*

| **Reference: Adzitey et al 2017** | **Rating** | **Description** |
| --- | --- | --- |
| 1. Was there a clear statement of the aims of the research? | Yes | "The aim of the study was to assess the knowledge and attitude of nurses in the Tamale Metropolis of Ghana towards KMC and its practice" |
| 2. Is a qualitative methodology appropriate? | Yes | Understanding knowledge and attitudes |
| 3. Was the research design appropriate to address the aims of the research? | Can’t tell | Not reported |
| 4. Was the recruitment strategy appropriate to the aims of the research? | Yes | Purposefully sampled facilities to be the main hospitals in the region, randomly sampled nurses working at the facilities |
| 5. Was the data collected in a way that addressed the research issue? | Yes | Hospital setting is appropriate research site to understand the research question, methods described, pre-tested structured questionnaire with open and closed ended questions |
| 6. Has the relationship between researcher and participants been adequately considered? | Can’t tell | Who conducted the interviews and relationship of researchers to participants not described |
| 7. Have ethical issues been taken into consideration? | No | No discussion of confidentiality or ethics approval |
| 8. Has the data analysis sufficiently rigorous? | No | While closed answer questions were described in descriptive statistics using SPSS, it is unclear how the qualitative analysis was conducted |
| 9. Is there a clear statement of findings? | Yes | Clearly states that many nurses have a positive attitude towards KMC but lack adequate knowledge |
| 10. How valuable is the research? | Yes | Summarizes barriers and facilitators, implications for practice |
| Comments on key limitations | Poor reporting in methodology on qualitative analysis and if ethical issues have been taken into consideration, cross-sectional representing a snapshot in time | |

| **Reference: Aliganyira et al 2014** | **Rating** | **Description** |
| --- | --- | --- |
| 1. Was there a clear statement of the aims of the research? | Yes | "Systematically measuring the scope and institutionalisation of KMC services and to describe barriers and facilitators to sustainable KMC services" |
| 2. Is a qualitative methodology appropriate? | Yes | Understanding stakeholder and health worker perspectives on implementation factors |
| 3. Was the research design appropriate to address the aims of the research? | Yes | Described that this mixed-methods design was used because it put a previously developed stages-of-change model |
| 4. Was the recruitment strategy appropriate to the aims of the research? | Yes | Sampled stakeholders, management teams, KMC focal persons and other staff - this is appropriate to understand a diversity of perspectives involved in implementing KMC |
| 5. Was the data collected in a way that addressed the research issue? | Yes | Hospital setting is appropriate research site to understand the research question, methods detailed with description of aspects covered |
| 6. Has the relationship between researcher and participants been adequately considered? | Can’t tell | Who conducted the interviews and relationship of researchers to participants not described |
| 7. Have ethical issues been taken into consideration? | Yes | Approval obtained by Ministry of Health, Uganda and John Hopkins School of Public Health, however confidentiality not explicitly reported |
| 8. Has the data analysis sufficiently rigorous? | No | While a scoring system was described, it was unclear how qualitative data was analyzed and how it contributed to the scoring system. |
| 9. Is there a clear statement of findings? | Yes | KMC services are not instituted with consistent levels of quality and are often dependent on private partner support |
| 10. How valuable is the research? | Yes | Summarizes barriers and facilitators, implications for practice |
| Comments on key limitations | Poor reporting on methodology of qualitative analyses, cross-sectional representing a snapshot in time | |

| **Reference: Bergh and Pattinson 2003** | **Rating** | **Description** |
| --- | --- | --- |
| 1. Was there a clear statement of the aims of the research? | Yes | “To develop a conceptual tool to assist healthcare workers and management in the implementation of a kangaroo mother care programme” |
| 2. Is a qualitative methodology appropriate? | Yes | “a qualitative approach aimed at mapping different processes and various alternatives in the practice of KMC” |
| 3. Was the research design appropriate to address the aims of the research? | Yes | Described action research that includes both quantitative and qualitative components |
| 4. Was the recruitment strategy appropriate to the aims of the research? | Yes | Hospital setting is appropriate research site to understand the research question, key informants included management, consultants, medical officers and nurses in charge as well as informal conversational interviews during on-site observations |
| 5. Was the data collected in a way that addressed the research issue? | Yes | Methods detailed with description of the interview guide |
| 6. Has the relationship between researcher and participants been adequately considered? | Yes | Described that data was collected by an outside researcher not employed in health facilities but familiar with them |
| 7. Have ethical issues been taken into consideration? | No | Ethical considerations not reported |
| 8. Has the data analysis sufficiently rigorous? | Yes | Interviews were recorded and analyzed with field notes, used thick description and conceptual maps |
| 9. Is there a clear statement of findings? | Can’t tell | Produced a diagram of main issues and table of questions under topic areas to evaluate but did not report a clear statement of the main issues |
| 10. How valuable is the research? | Yes | diagram is a starting point for discussions on the implementation of KMC |
| Comments on key limitations | Key issues inferred from the diagram but not explicitly summarized, cross-sectional representing a snapshot in time | |

| **Reference: Bergh et al 2014** | **Rating** | **Description** |
| --- | --- | --- |
| 1. Was there a clear statement of the aims of the research? | Yes | "To systematically evaluate the implementation status of facility-based kangaroo mother care services in four African countries: Malawi, Mali, Rwanda and Uganda.” |
| 2. Is a qualitative methodology appropriate? | Yes | Understanding stakeholder and health worker perspectives on implementation factors |
| 3. Was the research design appropriate to address the aims of the research? | Yes | Semi-structured interviews with key informants and observations contextualized the facility assessment findings |
| 4. Was the recruitment strategy appropriate to the aims of the research? | Can’t tell | Convenience sampling of health facilities, however, sampled stakeholders, management teams, KMC focal persons and other staff - this is appropriate to understand a diversity of perspectives involved in implementing KMC |
| 5. Was the data collected in a way that addressed the research issue? | Yes | Methods detailed with description of the interview guide, data collections tools available as online supplement and previously used in other studies |
| 6. Has the relationship between researcher and participants been adequately considered? | Can’t tell | "In each country a team of local assessors with clinical and/or training experience in KMC were trained in the use of the facility tool" - previous experience with these facilities unclear |
| 7. Have ethical issues been taken into consideration? | Yes | "The research protocol was approved by the Institutional Review Board of the Johns Hopkins Bloomberg School of Public Health (IRB no. 0004134). The ministries of health and all facility directors gave written per-mission for the study and key informants in facilities gave oral consent before being interviewed and showing" wards |
| 8. Has the data analysis sufficiently rigorous? | Yes | Detailed description of data analysis reported including process, thematic analysis |
| 9. Is there a clear statement of findings? | Yes | Produced a diagram of main issues and table of questions under topic areas to evaluate but did not report a clear statement of the main issues |
| 10. How valuable is the research? | Yes | Summarizes barriers and facilitators, implications for practice |
| Comments on key limitations | Convenience sampling of health facilities, lack of clarity about relationship between researchers and the study facilities, cross-sectional representing a snapshot in time | |

| **Reference: Chisenga et al 2015** | **Rating** | **Description** |
| --- | --- | --- |
| 1. Was there a clear statement of the aims of the research? | Yes | “To review experiences of mothers Kangaroo Mother Care at two hospitals of Bwaila and Zomba.” |
| 2. Is a qualitative methodology appropriate? | Yes | “qualitative design to review mothers' KMC experiences” |
| 3. Was the research design appropriate to address the aims of the research? | Yes | Described pre-tested interview questionnaire with open and closed questions. “Open-ended questions were included in the questionnaire to allow participants to identify variables not fore-seen by the researcher” |
| 4. Was the recruitment strategy appropriate to the aims of the research? | Yes | Hospital setting is appropriate research site to understand the research question, target population and eligibility criteria well defined, not clear on how many declined participation |
| 5. Was the data collected in a way that addressed the research issue? | Yes | Methods detailed with description of interview guide, questionnaire pretested in a pilot study and reviewed by a panel of experts, data collection procedure well described |
| 6. Has the relationship between researcher and participants been adequately considered? | Can’t tell | Who conducted the interviews and relationship of researchers to participants not described |
| 7. Have ethical issues been taken into consideration? | Yes | “Both verbal and written consent were obtained before the interview was conducted” and “Permission to conduct the study was sought from Kamuzu College of Nursing Research and Ethics Committee, College of Medicine Research and Ethics Committee and from the Hospital Directors of Bwaila and Zomba Central…” |
| 8. Has the data analysis sufficiently rigorous? | Yes | “Open-ended questions, such as those on demographic characteristics, awareness and knowledge of beneﬁts, attitudes towards KMC, normative beliefs regarding KMC and motivation to comply with KMC practice, were coded according to content and ranked in order of frequency”, theoretical framework also described (theory of reasoned action) |
| 9. Is there a clear statement of findings? | Yes | Mothers had a positive attitude towards KMC once fully aware of its beneﬁts but lack of counselling, long length of stay in hospital, lack of assistance with skin-to-skin contact, multiple roles of the mother, culture, lack of support, lack of motivation, not being comfortable with the kangaroo position, and stigma can affect continuation and compliance of KMC. |
| 10. How valuable is the research? | Yes | Summarizes barriers and facilitators, implications for practice |
| Comments on key limitations | Lack of clarity about relationship between researchers and the study facilities, cross-sectional representing a snapshot in time | |

| **Reference: Davidge 2009** | **Rating** | **Description** |
| --- | --- | --- |
| 1. Was there a clear statement of the aims of the research? | Can’t tell | “This article provides an insight into the need for and commencement of Kangaroo Mother Care  (KMC) in the neonatal intensive care unit at a tertiary hospital in KwaZulu Natal, South Africa.” –aims of the article are briefly highlighted but the statement is ambiguous |
| 2. Is a qualitative methodology appropriate? | Yes | A reflection to elaborate on lessons learned, though this is not explicitly descried in the article objective |
| 3. Was the research design appropriate to address the aims of the research? | Can’t tell | Lack of details on methods |
| 4. Was the recruitment strategy appropriate to the aims of the research? | Can’t tell | Lack of details on methods |
| 5. Was the data collected in a way that addressed the research issue? | Can’t tell | Lack of details on methods |
| 6. Has the relationship between researcher and participants been adequately considered? | No | Does not consider how positionality may have influenced the key informant reflection |
| 7. Have ethical issues been taken into consideration? | No | No discussion of ethics |
| 8. Has the data analysis sufficiently rigorous? | No | Lack of details on methods |
| 9. Is there a clear statement of findings? | Yes | Private hospitals that are well supplied with neonatal technology do not see the need to promote KMC, which is to the detriment of the health of preterm and low birthweight babies |
| 10. How valuable is the research? | Can’t tell | Does not explicitly describes implications for practice |
| Comments on key limitations | Lack of information about methods overall but some valuable insights from the researcher’s personal experiences. | |

| **Reference: Gondwe et al 2016** | **Rating** | **Description** |
| --- | --- | --- |
| 1. Was there a clear statement of the aims of the research? | Yes | “to explore the views of health stakeholders  about the care of preterm infants in health facilities and the existence of any policy protocol documents guiding the delivery of care to these infants” |
| 2. Is a qualitative methodology appropriate? | Yes | To understand views and perspectives |
| 3. Was the research design appropriate to address the aims of the research? | Yes | Described use of semi-structured interviews with open-ended questions to collect data |
| 4. Was the recruitment strategy appropriate to the aims of the research? | Yes | Used purposeful sampling to select health stakeholders in 10 health facilities and 5 health stakeholder  offices |
| 5. Was the data collected in a way that addressed the research issue? | Yes | Methods detailed with description of the interview guide |
| 6. Has the relationship between researcher and participants been adequately considered? | CD | Who conducted the interviews and relationship of researchers to participants not described |
| 7. Have ethical issues been taken into consideration? | Yes | “Ethical approval was obtained from the College of Medicine Research and Ethics Committee (COMREC) in Malawi. All participants received written and detailed verbal information about the aim of the study and the procedure of data collection during the time of booking appointments with interviewees.” |
| 8. Has the data analysis sufficiently rigorous? | Yes | Data analysis methods clearly described and methodology referenced |
| 9. Is there a clear statement of findings? | Yes | There is a need to improve health worker knowledge about preterm infant care |
| 10. How valuable is the research? | Yes | Summarizes barriers and facilitators, implications for practice |
| Comments on key limitations | Lack of clarity about relationship between researchers and the study facilities, cross-sectional representing a snapshot in time | |

| **Reference: Karbarami et al 2002** | **Rating** | **Description** |
| --- | --- | --- |
| 1. Was there a clear statement of the aims of the research? | Yes | “To document caregivers’ perceptions, experiences, concerns and constraints in relation to kangaroo care, both in hospital and in their community.” |
| 2. Is a qualitative methodology appropriate? | Yes | To understand perceptions and experiences |
| 3. Was the research design appropriate to address the aims of the research? | Yes | Focus group discussion were used to collect information about family perceptions |
| 4. Was the recruitment strategy appropriate to the aims of the research? | No | Described four focus group discussions with different groups of family members but unclear how many people were in each of the group, the process of recruitment and how family members were approached, and how many refused participation. Also bias may have been introduced by only interviewing mothers of infants who had survived. |
| 5. Was the data collected in a way that addressed the research issue? | Yes | Hospital setting is appropriate research site to understand the research question, FGD guide was developed by a senior social scientist in collaboration with a senior paediatrician |
| 6. Has the relationship between researcher and participants been adequately considered? | Can’t tell | Interviews conducted by a trained research nurse with prior experience conducting interviews. Described that the research nurse was not involved in KMC prior to the study but their relationship to the facility or positionality as a nurse |
| 7. Have ethical issues been taken into consideration? | No | No discussion of confidentiality or ethics approval |
| 8. Has the data analysis sufficiently rigorous? | No | Audio transcribed verbatim but overall lack of information on qualitative analysis |
| 9. Is there a clear statement of findings? | Yes | Mothers in this study were aware of the kangaroo care methods, its benefits and preferred it in comparison to incubator care but poor awareness among the community and there were training gaps among nursing staff |
| 10. How valuable is the research? | Yes | Compiled recommendations from mothers engaged in KMC |
| Comments on key limitations | Limited information on methods, lack of clarity about relationship between researchers and the study facilities, cross-sectional representing a snapshot in time | |

| **Reference: Kiwanuka et al 2017** | **Rating** | **Description** |
| --- | --- | --- |
| 1. Was there a clear statement of the aims of the research? | Yes | “To assess challenges facing mothers who practice kangaroo mother care method for  their preterm or low birth weight babies in Dar es Salaam.” |
| 2. Is a qualitative methodology appropriate? | Yes | To understand experiences |
| 3. Was the research design appropriate to address the aims of the research? | Yes | Structured interviews, focus group discussion and observation were used to collect information about mothers experiences |
| 4. Was the recruitment strategy appropriate to the aims of the research? | Yes | “ All women who practiced kangaroo care in the hospital KMC unit having preterm infants or low birth weight babies and were willing to participate in the study were interviewed” |
| 5. Was the data collected in a way that addressed the research issue? | Yes | Hospital setting is appropriate research site to understand the research question, interview guide pretested |
| 6. Has the relationship between researcher and participants been adequately considered? | No | Unclear who conducted the data collection |
| 7. Have ethical issues been taken into consideration? | Yes | Ethics approval reported and issues described in their consideration |
| 8. Has the data analysis sufficiently rigorous? | Can’t tell | Audio transcribed verbatim but overall lack of information on qualitative analysis beyond stating content analysis |
| 9. Is there a clear statement of findings? | Yes | Many women were not aware of this method making it hard for them to practice KMC effectively |
| 10. How valuable is the research? | Yes | Highlighted mothers perspectives on difficulty of facility-based KMC |
| Comments on key limitations | Limited information on data collection procedure, lack of clarity about relationship between researchers and the study facilities, cross-sectional representing a snapshot in time | |

| **Reference: Leonard and Mayers 2008** | **Rating** | **Description** |
| --- | --- | --- |
| 1. Was there a clear statement of the aims of the research? | Yes | “To explore parents’ lived experience of providing KC to their preterm infants in a tertiary hospital setting in Cape Town” |
| 2. Is a qualitative methodology appropriate? | Yes | “Phenomenology is appropriate for studies in which the research question aims to extract the “meaning or essence of an experience” (Morse, 1994:224) or “to describe a person’s lived e experience (phenomena)” (Van Manen, 1990:38).” |
| 3. Was the research design appropriate to address the aims of the research? | Yes | Described using in-depth interviews and observations, and referenced phenomenological interviewing style |
| 4. Was the recruitment strategy appropriate to the aims of the research? | Yes | “The study population comprised all parents who were actively involved in providing KC to their preterm infants at a tertiary hospital in Cape Town. Both parents were included in the study, since fathers have historically been under-represented… Information-rich cases were selected for in-depth study, since this allowed the central issues of the phenomenon (providing KC) to be uncovered…” |
| 5. Was the data collected in a way that addressed the research issue? | Can’t tell | Hospital setting is appropriate research site to understand the research question, interviews collected until data saturation. However, researchers note that interviews were conducted in English, which was not some participants’ home language. While interview methods described, unclear the process of observations. |
| 6. Has the relationship between researcher and participants been adequately considered? | Can’t tell | Who conducted the interviews and relationship of researchers to participants not described |
| 7. Have ethical issues been taken into consideration? | Yes | Ethical considerations and approvals for local ethic boards reported |
| 8. Has the data analysis sufficiently rigorous? | Can’t tell | Interviews were transcribed verbatim and process of analysis reported. Process of analyzing observations unclear. |
| 9. Is there a clear statement of findings? | Yes | Parents grappled with adapting to preterm birth and coping with previously unfamiliar kangaroo care. Health workers are a valuable resource of support. |
| 10. How valuable is the research? | Yes | Summarizes barriers and facilitators, implications for practice |
| Comments on key limitations | Interviews were conducted in English, which may have represented a language barrier for some, lack of clarity about relationship between researchers and the study facilities, cross-sectional representing a snapshot in time | |

| **Reference: Morgan et al 2018** | **Rating** | **Description** |
| --- | --- | --- |
| 1. Was there a clear statement of the aims of the research? | Yes | “We aimed to determine the proportion of admitted neonates meeting pro-posed instability criteria, assess the feasibility of providing KMC to unstable neonates, and evaluate the acceptability of this intervention to parents and providers at Jinja Regional Referral Hospital in Uganda” |
| 2. Is a qualitative methodology appropriate? | Yes | Qualitative methodology appropriate for evaluating acceptability of KMC |
| 3. Was the research design appropriate to address the aims of the research? | Yes | Described using semi-structured interviews, which is appropriate to understand acceptability of KMC for unstable neonates |
| 4. Was the recruitment strategy appropriate to the aims of the research? | Yes | Purposeful sampling of 20 key stakeholders (parents and providers) |
| 5. Was the data collected in a way that addressed the research issue? | Can’t tell | Hospital setting is appropriate research site to understand the research question. Noted that “the correct sample size is one that satisfactorily answers the research question” but did not discuss data saturation or how their sample size satisfactorily answers research question. Option for mothers and other family members to conduct interview in language of preference. The interview guide employed open-ended questions about a broad range of potential factors while allowing the interviewer to ask additional questions on emerging themes. |
| 6. Has the relationship between researcher and participants been adequately considered? | Can’t tell | “interviewer was a local woman who spoke the local language” but unclear the relationship between researcher and participants |
| 7. Have ethical issues been taken into consideration? | Yes | Ethical considerations and approvals for local ethic boards reported |
| 8. Has the data analysis sufficiently rigorous? | Yes | Process of thematic analysis well described |
| 9. Is there a clear statement of findings? | Yes | KMC for unstable neonates weighing ≤2000g was feasible and acceptable |
| 10. How valuable is the research? | Yes | Summarizes barriers and facilitators, implications for practice |
| Comments on key limitations | A mixed-method study that triangulated a retrospective chart review, exploratory feasibility study and qualitative acceptability study. For the interests of this review, focused on the qualitative acceptability study as it reported barriers and facilitators. Some lack of clarity about relationship between data collectors and the study facilities, cross-sectional representing a snapshot in time | |

| **Reference: Reddy and McInerney 2007** | **Rating** | **Description** |
| --- | --- | --- |
| 1. Was there a clear statement of the aims of the research? | Yes | “To explore the perceptions of mothers for the preparation and experience of KMC and to describe the experiences of the mothers who had implemented KMC” |
| 2. Is a qualitative methodology appropriate? | Yes | Understand perceptions and experiences |
| 3. Was the research design appropriate to address the aims of the research? | Yes | In-depth interviews in the phenomenology approach |
| 4. Was the recruitment strategy appropriate to the aims of the research? | Yes | “A purposeful theoretical sampling technique was used. The sample was drawn from the mothers whose babies had been admitted to the neonatal unit of the regional hospital and who subsequently went on to practice KMC.” |
| 5. Was the data collected in a way that addressed the research issue? | Can’t tell | Hospital setting is appropriate research site to understand the research question, sampling continued until saturation of data was achieved. However, language requirement that mothers had to speak English. |
| 6. Has the relationship between researcher and participants been adequately considered? | Can’t tell | Who conducted the interviews and relationship of researchers to participants not described |
| 7. Have ethical issues been taken into consideration? | Yes | Researchers described ethical considerations and how they protected confidentially |
| 8. Has the data analysis sufficiently rigorous? | Yes | Reported that interviews were transcribed verbatim and analyzed using Morse’s cognitive process of data analysis. |
| 9. Is there a clear statement of findings? | Yes | KMC was new to most of the mothers and were fearful at first and nurses were important in building a supportive environment for the mothers practicing KMC |
| 10. How valuable is the research? | Yes | Summarizes barriers and facilitators, implications for practice |
| Comments on key limitations | Interviews were conducted in English, which may have represented a language barrier for some, lack of clarity about relationship between researchers and the study facilities, cross-sectional representing a snapshot in time | |

| **Reference: Söderbäck and Erlandsson 2012** | **Rating** | **Description** |
| --- | --- | --- |
| 1. Was there a clear statement of the aims of the research? | Yes | “To describe events at a Mozambican neonatal unit and mothers’ experiences of going through admission, passing from an intensive care ward to a nursery ward with their premature baby, and then undergoing KMC training before early discharge home” |
| 2. Is a qualitative methodology appropriate? | Yes | Understand perceptions and experiences |
| 3. Was the research design appropriate to address the aims of the research? | Yes | In-depth interviews and naturalistic observations in the phenomenology approach |
| 4. Was the recruitment strategy appropriate to the aims of the research? | Can’t tell | A purposive sample of 41 mothers admitted with their premature baby/babies was invited and all agreed to participate though unclear why a sample of 41 was selected. |
| 5. Was the data collected in a way that addressed the research issue? | Can’t tell | Hospital setting is appropriate research site to understand the research question, it is unclear whether two days a week for a month was enough time for naturalistic observations |
| 6. Has the relationship between researcher and participants been adequately considered? | Can’t tell | “the first author is a paediatric nurse who  gained knowledge about Mozambican culture while living there for several years and that the second author, who contributed to the analysis and the writing of the manuscript, is also experienced in KMC” reported as a strength of the study but relationship between the researchers and participants remain unclear. |
| 7. Have ethical issues been taken into consideration? | No | Ethics and confidentiality not discussed |
| 8. Has the data analysis sufficiently rigorous? | Can’t tell | Research assistant wrote mothers’ responses to open questions in verbatim as possible and analysed using manifest content analysis, unclear how observations were analyzed |
| 9. Is there a clear statement of findings? | Yes | Highlights complexity of KMC and finds that the hierarchical hospital organization was a barrier to mothers getting the support they needed to implement KMC |
| 10. How valuable is the research? | Can’t tell | Barriers and facilitators summarized but policy implications and implementation strategies are less explicitly stated |
| Comments on key limitations | Lack of clarity on the positionality of the researchers and the methods/analysis of the observations, implications for policy not clearly described, cross-sectional representing a snapshot in time | |

| **Reference: ten Ham, Minnie and van der Walt 2016** | **Rating** | **Description** |
| --- | --- | --- |
| 1. Was there a clear statement of the aims of the research? | Yes | “To explore and describe the perspectives of health professionals on the requirements for the rolling-out process of KMC as a best practice in South Africa” |
| 2. Is a qualitative methodology appropriate? | Yes | Exploring perspectives |
| 3. Was the research design appropriate to address the aims of the research? | Yes | Semi-structured interviews with key informants to understand process of implementation |
| 4. Was the recruitment strategy appropriate to the aims of the research? | Yes | A combination of purposive and snowball sampling was used to identify and recruit key informants that were involved in the implementation and rolling-out process of KMC. Sampling framework reported. |
| 5. Was the data collected in a way that addressed the research issue? | Yes | Semi-structured interview guide reported and well described |
| 6. Has the relationship between researcher and participants been adequately considered? | Can’t tell | “XXX conducted the interviews” - unclear who XXX was and their connection to the participants |
| 7. Have ethical issues been taken into consideration? | Yes | Ethical considerations reported |
| 8. Has the data analysis sufficiently rigorous? | Yes | Transcripts were shared with participants with cross-checking and data analyzed using content analysis |
| 9. Is there a clear statement of findings? | Yes | Requirements for roll-out exist at all levels of the health care system |
| 10. How valuable is the research? | Yes | Summarizes barriers and facilitators, implications for practice |
| Comments on key limitations | Unclear who researcher “XXX” was and their relationship with participants, cross-sectional representing a snapshot in time | |

**CASE SERIES (NIH quality assessment tools)**

| **Reference** | **1** | **2** | **3** | **4** | **5** | **6** | **7** | **8** | **9** | **Rating** |
| --- | --- | --- | --- | --- | --- | --- | --- | --- | --- | --- |
| Watkins et al 2018 | Y | Y | CD | CD | Y | Y | Y | Y | Y | Fair |

*Y – Yes, N – No, CD – Cannot determine/ cannot tell, NR – not reported, NA – not applicable*

| **Reference:** **Watkins et al 2018** | **Rating** | **Description** |
| --- | --- | --- |
| 1. Was the study question or objective clearly stated? | Yes | “To quantify the daily duration of SSC  over the first week of life and to compare these findings with the data collected on SSC continuity by the existing literature” |
| 2. Was the study population clearly and fully described, including a case definition? | Yes | Reported an inclusion criteria of: inborn at Jinja Regional Referral Hospital, born weighing up to  2000 g, deemed clinically stable by the admitting clinician and the mother was willing and able to participate in KMC |
| 3. Were the cases consecutive? | Cannot determine | Reported that 12 of the 68 infants admitted during the study period met the inclusion criteria but unclear if these cases were consecutive |
| 4. Were the subjects comparable? | Cannot determine | Reported that some mothers and/or infants were ill and overall mean birthweight and gestational age but description of participants not reported separately |
| 5. Was the intervention clearly described? | Yes | Skin-to-skin contact clearly described |
| 6. Were the outcome measures clearly defined, valid, reliable, and implemented consistently across all study participants? | Yes | Cumulative daily duration of SSC over the first week of life, 20 hours a day was the cut-off for continuous |
| 7. Was the length of follow-up adequate? | Yes | “continuous observation of infants began as soon as possible after birth and continued until the  end of day of life seven, discharge or death, whichever came first” |
| 8. Were the statistical methods well-described? | Yes | Described software to run analyses and procedures. Did not do survival analysis due to small sample size. |
| 9. Were the results well-described? | Yes | All primary and secondary outcomes described in methods were reported |
| Comments on key limitations | Lack of clarity on case characteristics | |

**OBSERVATIONAL COHORT/ CROSS-SECTIONAL SURVEY (NIH quality assessment tools)**

| **Reference** | **1** | **2** | **3** | **4** | **5** | **6** | **7** | **8** | **9** | **10** | **11** | **12** | **13** | **14** | **Rating** |
| --- | --- | --- | --- | --- | --- | --- | --- | --- | --- | --- | --- | --- | --- | --- | --- |
| Bergh et al 2012 | Y | Y | Y | Y | NR | Y | CD | NA | Y | NA | Y | N | Y | N | Fair |
| Bergh et al 2013 | Y | Y | Y | Y | NR | Y | Y | NA | Y | NA | Y | N | Y | N | Fair |
| Chavula et al 2017 | Y | Y | Y | Y | Y | N | NA | CD | Y | N | Y | N | NA | N | Fair |
| Feucht et al 2015 | Y | Y | Y | Y | Y | Y | Y | CD | CD | Y | Y | N | Y | N | Fair |
| Kambarami, Chidede and Kowo 1999 | Y | Y | CD | Y | NR | N | NA | N | CD | N | CD | CD | NA | Y | Poor |
| Kampekete, Ngoma and Masumo 2018 | Y | Y | Y | Y | Y | N | NA | Y | Y | N | Y | N | NA | N | Fair |
| Lincetto, Nazir and Cattaneo 2000 | Y | Y | Y | Y | N | Y | CD | Y | CD | CD | CD | N | NR | N | Poor |
| Namazzi et al 2015 | Y | Y | Y | Y | CD | Y | Y | CD | CD | Y | Y | N | Y | N | Fair |
| Onubogu and Okoh 2016 | Y | Y | Y | Y | NR | N | NA | Y | N | N | N | N | NA | N | Poor |
| Solomons and Rosant 2012 | Y | Y | Y | Y | N | N | NA | Y | CD | N | N | N | NA | N | Poor |
| Weldearegay et al 2019 | Y | Y | Y | Y | Y | N | NA | Y | Y | N | Y | N | NA | Y | Fair |

*Y – Yes, N – No, CD – Cannot determine/ cannot tell, NR – not reported, NA – not applicable*

| **Reference:** **Bergh et al 2012** | **Rating** | **Description** |
| --- | --- | --- |
| 1. Was the research question or objective in this paper clearly stated? | Yes | “to describe the processes followed in the translation of the findings from implementing KMC in South Africa to a contextualised, practical application in Ghana and the results of the monitoring of the progress made with the implementation of KMC" |
| 2. Was the study population clearly specified and defined? | Yes | Hospitals in four regions in Ghana |
| 3. Was the participation rate of eligible persons at least 50%? | Yes | All 38 hospitals in the four regions participated |
| 4. Were all the subjects selected or recruited from the same or similar populations (including the same time period)? Were inclusion and exclusion criteria for being in the study prespecified and applied uniformly to all participants? | Yes | Recruited at same time and place |
| 5. Was a sample size justification, power description, or variance and effect estimates provided? | Not reported | All hospitals in the region were included but did not discuss sample size justifications for why four out of the 10 regions in Ghana were selected |
| 6. For the analyses in this paper, were the exposure(s) of interest measured prior to the outcome(s) being measured? | Yes | Implementation of KMC taken as non-existent before implementation |
| 7. Was the timeframe sufficient so that one could reasonably expect to see an association between exposure and outcome if it existed? | Cannot determine | One year after implementation - may not be long enough to see behavioural change or sustainability of practices |
| 8. For exposures that can vary in amount or level, did the study examine different levels of the exposure as related to the outcome (e.g., categories of exposure, or exposure measured as continuous variable)? | NA | NA |
| 9. Were the exposure measures (independent variables) clearly defined, valid, reliable, and implemented consistently across all study participants? | Yes | Intervention methodology well described |
| 10. Was the exposure(s) assessed more than once over time? | NA | NA |
| 11. Were the outcome measures (dependent variables) clearly defined, valid, reliable, and implemented consistently across all study participants? | Yes | Assessment based on a validated model with established scoring system |
| 12. Were the outcome assessors blinded to the exposure status of participants? | No | Study design did not allow for blinding |
| 13. Was loss to follow-up after baseline 20% or less? | Yes | No health facilities dropped out |
| 14. Were key potential confounding variables measured and adjusted statistically for their impact on the relationship between exposure(s) and outcome(s)? | No | Descriptive summaries only |
| Comments on key limitations | Unclear if one year is long enough to evaluate sustainable implementation, study design did not allow for blinding since all hospitals received the intervention, all hospitals in the region were included and there were no withdrawals, assessment based on a validated tool | |

| **Reference:** **Bergh et al 2013** | **Rating** | **Description** |
| --- | --- | --- |
| 1. Was the research question or objective in this paper clearly stated? | Yes | "To measure progress with the implementation of kangaroo mother care (KMC) for low birth-weight (LBW) infants at a health systems level." |
| 2. Was the study population clearly specified and defined? | Yes | Hospitals in four regions in Ghana |
| 3. Was the participation rate of eligible persons at least 50%? | Yes | All 38 hospitals in the four regions participated |
| 4. Were all the subjects selected or recruited from the same or similar populations (including the same time period)? Were inclusion and exclusion criteria for being in the study prespecified and applied uniformly to all participants? | Yes | Recruited at same time and place |
| 5. Was a sample size justification, power description, or variance and effect estimates provided? | Not reported | All hospitals in the region were included but did not discuss sample size justifications for why four out of the 10 regions in Ghana were selected |
| 6. For the analyses in this paper, were the exposure(s) of interest measured prior to the outcome(s) being measured? | Yes | Implementation of KMC taken as non-existent before implementation |
| 7. Was the timeframe sufficient so that one could reasonably expect to see an association between exposure and outcome if it existed? | Yes | One year after end of project |
| 8. For exposures that can vary in amount or level, did the study examine different levels of the exposure as related to the outcome (e.g., categories of exposure, or exposure measured as continuous variable)? | NA | NA |
| 9. Were the exposure measures (independent variables) clearly defined, valid, reliable, and implemented consistently across all study participants? | Yes | Intervention methodology well described |
| 10. Was the exposure(s) assessed more than once over time? | NA | NA |
| 11. Were the outcome measures (dependent variables) clearly defined, valid, reliable, and implemented consistently across all study participants? | Yes | A standardised progress-monitoring instrument was used to collect quantitative and qualitative data for each hospital |
| 12. Were the outcome assessors blinded to the exposure status of participants? | No | Study design did not allow for blinding |
| 13. Was loss to follow-up after baseline 20% or less? | Yes | No health facilities dropped out |
| 14. Were key potential confounding variables measured and adjusted statistically for their impact on the relationship between exposure(s) and outcome(s)? | No | Descriptive summaries only |
| Comments on key limitations | Follow up to the Bergh et al 2012 study, study design did not allow for blinding since all hospitals received the intervention, all hospitals in the region were included and there were no withdrawals, assessment based on a validated tool | |

| **Reference:** **Chavula et al 2017** | **Rating** | **Description** |
| --- | --- | --- |
| 1. Was the research question or objective in this paper clearly stated? | Yes | “to describe the processes followed in the translation of the findings from implementing KMC in South Africa to a contextualised, practical application in Ghana and the results of the monitoring of the progress made with the implementation of KMC" |
| 2. Was the study population clearly specified and defined? | Yes | All hospitals in Malawi |
| 3. Was the participation rate of eligible persons at least 50%? | Yes | All participated |
| 4. Were all the subjects selected or recruited from the same or similar populations (including the same time period)? Were inclusion and exclusion criteria for being in the study prespecified and applied uniformly to all participants? | Yes | All hospitals as part of the Ministry of Health in Malawi 2014 nationwide assessment of EmONC services |
| 5. Was a sample size justification, power description, or variance and effect estimates provided? | Yes | Sample covered all hospitals |
| 6. For the analyses in this paper, were the exposure(s) of interest measured prior to the outcome(s) being measured? | No | Cross-sectional survey |
| 7. Was the timeframe sufficient so that one could reasonably expect to see an association between exposure and outcome if it existed? | NA | Cross-sectional survey |
| 8. For exposures that can vary in amount or level, did the study examine different levels of the exposure as related to the outcome (e.g., categories of exposure, or exposure measured as continuous variable)? | Cannot determine | May look to be binary yes/no availability indicators but unclear |
| 9. Were the exposure measures (independent variables) clearly defined, valid, reliable, and implemented consistently across all study participants? | Yes | Used WHO Service Availability and Readiness Assessment (SARA) domains |
| 10. Was the exposure(s) assessed more than once over time? | No | Cross-sectional survey |
| 11. Were the outcome measures (dependent variables) clearly defined, valid, reliable, and implemented consistently across all study participants? | Yes | Used a standard international definitions of KMC. Conducted a 12–month register review and calculated a facility KMC initiation rate by dividing the reported number of babies initiated on KMC by the number of live births at each facility. KMC defined using a standard international definitions of KMC. |
| 12. Were the outcome assessors blinded to the exposure status of participants? | No | Facility evaluation completed by same team of data collectors as those who did the registry review |
| 13. Was loss to follow-up after baseline 20% or less? | NA | Cross-sectional survey |
| 14. Were key potential confounding variables measured and adjusted statistically for their impact on the relationship between exposure(s) and outcome(s)? | No | Descriptive summaries only |
| Comments on key limitations | A cross-sectional survey of all hospitals in Malawi. Used a validated survey instrument conducted by a trained team of data collectors. Definitions of indicators for Kangaroo Mother care (KMC) service readiness and KMC operational status clearly described. Outcome assessed using retrospective hospital registry review so blinding of outcome assessors was less critical. | |

| **Reference:** **Feucht et al 2015** | **Rating** | **Description** |
| --- | --- | --- |
| 1. Was the research question or objective in this paper clearly stated? | Yes | “This article describes a quality improvement process undertaken in the Tshwane District, Gauteng Province, in which strengthening of KMC was used as an entry point for the improvement of neonatal care under the supervision of the DCST” |
| 2. Was the study population clearly specified and defined? | Yes | Health facilities in Tshwane District |
| 3. Was the participation rate of eligible persons at least 50%? | Yes | All eight health facilities in the district participated (two central, one tertiary, one regional and four district hospitals) |
| 4. Were all the subjects selected or recruited from the same or similar populations (including the same time period)? Were inclusion and exclusion criteria for being in the study prespecified and applied uniformly to all participants? | Yes | Health facilities in Tshwane District |
| 5. Was a sample size justification, power description, or variance and effect estimates provided? | Yes | Sample covered all hospitals |
| 6. For the analyses in this paper, were the exposure(s) of interest measured prior to the outcome(s) being measured? | Yes | District clinical specialist teams were formed and participated in three workshops before the hospital walk-through and final workshop to compile lessons learned |
| 7. Was the timeframe sufficient so that one could reasonably expect to see an association between exposure and outcome if it existed? | Yes | Two years (2013-2015) is likely a sufficient time to understand process evaluation indicators of district clinical specialist teams |
| 8. For exposures that can vary in amount or level, did the study examine different levels of the exposure as related to the outcome (e.g., categories of exposure, or exposure measured as continuous variable)? | Cannot determine | Lack of information on how exposure indicators were measured |
| 9. Were the exposure measures (independent variables) clearly defined, valid, reliable, and implemented consistently across all study participants? | Cannot determine | It is unclear whether district clinical specialist teams were uniformly implemented across the timeframe and district as well as the composition of the workshops |
| 10. Was the exposure(s) assessed more than once over time? | Yes | Each workshop comprised included feedback on each hospital’s KMC implementation process including achievements and challenges |
| 11. Were the outcome measures (dependent variables) clearly defined, valid, reliable, and implemented consistently across all study participants? | Yes | A standard progress monitoring tool validated in previous studies was used for evaluating KMC implementation in each hospital |
| 12. Were the outcome assessors blinded to the exposure status of participants? | No | Study design did not allow for blinding |
| 13. Was loss to follow-up after baseline 20% or less? | Yes | No health facilities dropped out |
| 14. Were key potential confounding variables measured and adjusted statistically for their impact on the relationship between exposure(s) and outcome(s)? | No | Descriptive summaries only |
| Comments on key limitations | Two years is likely long enough to understand lessons learned regarding district clinical specialist teams, study design did not allow for blinding since all hospitals received the intervention, all hospitals in the region were included and there were no withdrawals, assessment based on a validated tool | |

| **Reference:** **Kambarami, Chidede and Kowo 1999** | **Rating** | **Description** |
| --- | --- | --- |
| 1. Was the research question or objective in this paper clearly stated? | Yes | “To describe the experience in a newly established Kangaroo Care Unit (KCU) at a tertiary level hospital and to identify factors associated with poor outcome in this unit." |
| 2. Was the study population clearly specified and defined? | Yes | Mothers admitted to the Kangaroo Care Unit at Harare Central Hospital, Zimbabwe and their well preterm infants |
| 3. Was the participation rate of eligible persons at least 50%? | Cannot determine | “The mothers in this study were fully informed about this method of care and no mother was sent to the KCU against her wish.” Unclear if any refused. |
| 4. Were all the subjects selected or recruited from the same or similar populations (including the same time period)? Were inclusion and exclusion criteria for being in the study prespecified and applied uniformly to all participants? | Yes | Study subjects were all infants admitted to the KCU and their mothers. Well preterm infants who were able to suck and weighed less than 2000g were identified during ward rounds in the NNU and sent to the KCU with their mothers if there were available beds. Preterm infants with congenital malformations or who were recovering from surgery or whose mothers were ill or twins were not sent to the KCU and were therefore excluded from the study. |
| 5. Was a sample size justification, power description, or variance and effect estimates provided? | Not reported | Sample size justification not reported |
| 6. For the analyses in this paper, were the exposure(s) of interest measured prior to the outcome(s) being measured? | No | Cross-sectional study |
| 7. Was the timeframe sufficient so that one could reasonably expect to see an association between exposure and outcome if it existed? | NA | Cross-sectional survey |
| 8. For exposures that can vary in amount or level, did the study examine different levels of the exposure as related to the outcome (e.g., categories of exposure, or exposure measured as continuous variable)? | No | Categories of exposure were described as binary indicators (see table 1) |
| 9. Were the exposure measures (independent variables) clearly defined, valid, reliable, and implemented consistently across all study participants? | Cannot determine | Clinical indicators documented by a trained research midwife but unclear the timing of data collection and definitions |
| 10. Was the exposure(s) assessed more than once over time? | No | Cross-sectional study |
| 11. Were the outcome measures (dependent variables) clearly defined, valid, reliable, and implemented consistently across all study participants? | Cannot determine | Method of data collection and definition of clinical indicators not well reported in methods |
| 12. Were the outcome assessors blinded to the exposure status of participants? | Cannot determine | Unclear how outcome data collection was conducted |
| 13. Was loss to follow-up after baseline 20% or less? | NA | Cross-sectional survey |
| 14. Were key potential confounding variables measured and adjusted statistically for their impact on the relationship between exposure(s) and outcome(s)? | Yes | Multivariate analyses were conducted to adjust for factors that were significant in univariate analysis |
| Comments on key limitations | A cross-sectional study with lack of clarity on refusal rate of eligible participants and methods of data collection for outcome measures. Multivariate analyses were conducted to adjust for potential confounders. | |

| **Reference:** **Kampekete, Ngoma and Masumo 2018** | **Rating** | **Description** |
| --- | --- | --- |
| 1. Was the research question or objective in this paper clearly stated? | Yes | “To investigate factors that influence the acceptance of KMC in the care of premature babies at University Teaching Hospital, Lusaka, Zambia." |
| 2. Was the study population clearly specified and defined? | Yes | “The target population for this study were mothers who were admitted in the KMC unit at University Teaching Hospital because they had premature babies weighing less than 2000 g. The key informants were health professionals who dealt with management issues regarding KMC at University Teaching Hospital.” |
| 3. Was the participation rate of eligible persons at least 50%? | Yes | In a span of six months, 131 babies were admitted with approximately half over the three study months and 60 were purposively recruited into the study |
| 4. Were all the subjects selected or recruited from the same or similar populations (including the same time period)? Were inclusion and exclusion criteria for being in the study prespecified and applied uniformly to all participants? | Yes | Postnatal mothers admitted with premature babies. See study population for more information (question 2). |
| 5. Was a sample size justification, power description, or variance and effect estimates provided? | Yes | Sample size calculations reported |
| 6. For the analyses in this paper, were the exposure(s) of interest measured prior to the outcome(s) being measured? | No | Cross-sectional study |
| 7. Was the timeframe sufficient so that one could reasonably expect to see an association between exposure and outcome if it existed? | NA | Cross-sectional study |
| 8. For exposures that can vary in amount or level, did the study examine different levels of the exposure as related to the outcome (e.g., categories of exposure, or exposure measured as continuous variable)? | Yes | Different levels available for responses where appropriate |
| 9. Were the exposure measures (independent variables) clearly defined, valid, reliable, and implemented consistently across all study participants? | Yes | Questionnaire was adapted from other similar studies (Blomqvist et al, 2012; MCHIP, 2012; Solomons and Rosant, 2012; Chisenga et al, 2015) |
| 10. Was the exposure(s) assessed more than once over time? | No | Cross-sectional study |
| 11. Were the outcome measures (dependent variables) clearly defined, valid, reliable, and implemented consistently across all study participants? | Yes | Questionnaire was adapted from other similar studies (Blomqvist et al, 2012; MCHIP, 2012; Solomons and Rosant, 2012; Chisenga et al, 2015) |
| 12. Were the outcome assessors blinded to the exposure status of participants? | No | Study design did not allow for blinding |
| 13. Was loss to follow-up after baseline 20% or less? | NA | Cross-sectional survey |
| 14. Were key potential confounding variables measured and adjusted statistically for their impact on the relationship between exposure(s) and outcome(s)? | No | Descriptive summaries only |
| Comments on key limitations | A cross-sectional study with qualitative elements that complimented quantitative results, which were the focus of the study. Study design did not allow for blinding and no adjustment for potential confounding. Clear description of study population, intervention and data collection questionnaire was adapted from previous similar studies. | |

| **Reference:** **Lincetto, Nazir and Cattaneo 2000** | **Rating** | **Description** |
| --- | --- | --- |
| 1. Was the research question or objective in this paper clearly stated? | Yes | “describes how KMC was introduced at Quelimane Provincial Hospital and presents the results of three months of full monitoring after six months of gradual but steady implementation |
| 2. Was the study population clearly specified and defined? | Yes | LBWI admitted at Quelimane Provincial Hospital over three months |
| 3. Was the participation rate of eligible persons at least 50%? | Yes | All 32 LBWI admitted during the study |
| 4. Were all the subjects selected or recruited from the same or similar populations (including the same time period)? Were inclusion and exclusion criteria for being in the study prespecified and applied uniformly to all participants? | Yes | LBWI admitted at Quelimane Provincial Hospital over three months |
| 5. Was a sample size justification, power description, or variance and effect estimates provided? | No | Data collection was stopped after three months with 32 LBWI. Study planned for 60-100 participants over the course of six months. |
| 6. For the analyses in this paper, were the exposure(s) of interest measured prior to the outcome(s) being measured? | Yes | KMC or non-KMC infants |
| 7. Was the timeframe sufficient so that one could reasonably expect to see an association between exposure and outcome if it existed? | Cannot determine | Measured survival in the 24 hours after admission and overall survival to discharge. Six months may be a short length of time for implementing. |
| 8. For exposures that can vary in amount or level, did the study examine different levels of the exposure as related to the outcome (e.g., categories of exposure, or exposure measured as continuous variable)? | Yes | Continuous variables summarized as mean (SD) or categorized with multiple levels |
| 9. Were the exposure measures (independent variables) clearly defined, valid, reliable, and implemented consistently across all study participants? | Cannot determine | Unclear if the comparison between KMC or non-KMC infants was planned for the study as some infants not receiving KMC appears accidental |
| 10. Was the exposure(s) assessed more than once over time? | Cannot determine | Infants were monitored during KMC but unclear if compliance was measured |
| 11. Were the outcome measures (dependent variables) clearly defined, valid, reliable, and implemented consistently across all study participants? | Cannot determine | Survival outcomes not clearly defined in methods. Data collection and analysis of implementation factors is unclear. |
| 12. Were the outcome assessors blinded to the exposure status of participants? | No | Study design did not allow for blinding |
| 13. Was loss to follow-up after baseline 20% or less? | Not reported | Loss to follow up not reported and it is unclear whether some mothers self-discharged before medical advice |
| 14. Were key potential confounding variables measured and adjusted statistically for their impact on the relationship between exposure(s) and outcome(s)? | No | Descriptive summaries only |
| Comments on key limitations | Small sample size and short follow-up for implementation study. Lack of information on methods and results. | |

| **Reference:** **Namazzi et al 2015** | **Rating** | **Description** |
| --- | --- | --- |
| 1. Was the research question or objective in this paper clearly stated? | Yes | “describes the health systems strengthening process used to improve quality of care across 20 health facilities in rural eastern Uganda, and assesses its effect on the outcome of high-risk newborn babies” |
| 2. Was the study population clearly specified and defined? | Yes | All health facilities within the district where the UNEST randomized controlled trial was being conducted |
| 3. Was the participation rate of eligible persons at least 50%? | Yes | All health facilities enrolled |
| 4. Were all the subjects selected or recruited from the same or similar populations (including the same time period)? Were inclusion and exclusion criteria for being in the study prespecified and applied uniformly to all participants? | Yes | Recruited at same time and place |
| 5. Was a sample size justification, power description, or variance and effect estimates provided? | Cannot determine | All 20 health facilities covering the whole district though KMC was only implemented at one facility (district hospital) |
| 6. For the analyses in this paper, were the exposure(s) of interest measured prior to the outcome(s) being measured? | Yes | Baseline data were extracted for the 2 years prior to study |
| 7. Was the timeframe sufficient so that one could reasonably expect to see an association between exposure and outcome if it existed? | Yes | Two years |
| 8. For exposures that can vary in amount or level, did the study examine different levels of the exposure as related to the outcome (e.g., categories of exposure, or exposure measured as continuous variable)? | Cannot determine | Insufficient information described |
| 9. Were the exposure measures (independent variables) clearly defined, valid, reliable, and implemented consistently across all study participants? | Cannot determine | Indicators measured using a structured tool based on the national standards for newborn healthcare services but were not clearly defined in methods |
| 10. Was the exposure(s) assessed more than once over time? | Yes | Quantitative data were obtained quarterly |
| 11. Were the outcome measures (dependent variables) clearly defined, valid, reliable, and implemented consistently across all study participants? | Yes | Data on the number of deliveries, stillbirths, neonatal deaths, patients discharged, as well as health worker training and turnover were collected from hospital registries |
| 12. Were the outcome assessors blinded to the exposure status of participants? | No | Study design did not allow for blinding |
| 13. Was loss to follow-up after baseline 20% or less? | Yes | No health facilities lost to follow up |
| 14. Were key potential confounding variables measured and adjusted statistically for their impact on the relationship between exposure(s) and outcome(s)? | No | Descriptive summaries only |
| Comments on key limitations | Health systems strengthening at facilities alongside a randomized controlled trial of community-level interventions. KMC was only implemented at one facility. Lack of clarity on indicators and descriptive summaries only. | |

| **Reference:** **Onubogu and Okoh 2016** | **Rating** | **Description** |
| --- | --- | --- |
| 1. Was the research question or objective in this paper clearly stated? | Yes | “The aim of this study was to determine the proportion of Nigerian health workers rendering paediatric care who practice KMC in their institution, and identify some challenges affecting the practice of KMC in Nigerian health institutions" |
| 2. Was the study population clearly specified and defined? | Yes | participants at 45th annual scientific conference of the Paediatric Association of Nigeria was conducted |
| 3. Was the participation rate of eligible persons at least 50%? | Yes | response rate of 70.4% |
| 4. Were all the subjects selected or recruited from the same or similar populations (including the same time period)? Were inclusion and exclusion criteria for being in the study prespecified and applied uniformly to all participants? | Yes | All recruited at a conference, all health providers attending |
| 5. Was a sample size justification, power description, or variance and effect estimates provided? | Not reported | Sample size justification not reported |
| 6. For the analyses in this paper, were the exposure(s) of interest measured prior to the outcome(s) being measured? | No | Cross-sectional survey |
| 7. Was the timeframe sufficient so that one could reasonably expect to see an association between exposure and outcome if it existed? | NA | Cross-sectional study |
| 8. For exposures that can vary in amount or level, did the study examine different levels of the exposure as related to the outcome (e.g., categories of exposure, or exposure measured as continuous variable)? | Yes | Indicators given in levels where appropriate |
| 9. Were the exposure measures (independent variables) clearly defined, valid, reliable, and implemented consistently across all study participants? | No | Indicators listed in methods but not defined. Unclear how the questionnaire was developed |
| 10. Was the exposure(s) assessed more than once over time? | No | Cross-sectional survey |
| 11. Were the outcome measures (dependent variables) clearly defined, valid, reliable, and implemented consistently across all study participants? | No | Lack of clarity on outcome measure definitions and nature, such as whether reasons for not practicing KMC in their facilities was predefined for selection or open answer. Poor reporting of results including some information only available in hard to read graphs and not summarized in text. |
| 12. Were the outcome assessors blinded to the exposure status of participants? | No | Study design did not allow for blinding |
| 13. Was loss to follow-up after baseline 20% or less? | NA | Cross-sectional survey |
| 14. Were key potential confounding variables measured and adjusted statistically for their impact on the relationship between exposure(s) and outcome(s)? | No | Descriptive summaries only |
| Comments on key limitations | Cross-sectional study with some lack of clarity in methods and reporting of results. | |

| **Reference:** **Solomons and Rosant 2012** | **Rating** | **Description** |
| --- | --- | --- |
| 1. Was the research question or objective in this paper clearly stated? | Yes | “to determine the knowledge and attitude of nursing staff and mothers towards kangaroo mother care (KMC) in the eastern sub-district of Cape Town" |
| 2. Was the study population clearly specified and defined? | Yes | Helderberg District Hospital (HDH), Somerset West |
| 3. Was the participation rate of eligible persons at least 50%? | Yes | 30 of 32 eligible mothers interviewed |
| 4. Were all the subjects selected or recruited from the same or similar populations (including the same time period)? Were inclusion and exclusion criteria for being in the study prespecified and applied uniformly to all participants? | Yes | LBW (< 2 500 g) infants delivered at HDH between 24 January 2008 to 17 March 2008 |
| 5. Was a sample size justification, power description, or variance and effect estimates provided? | No | Sample size justification not reported |
| 6. For the analyses in this paper, were the exposure(s) of interest measured prior to the outcome(s) being measured? | No | Cross-sectional survey |
| 7. Was the timeframe sufficient so that one could reasonably expect to see an association between exposure and outcome if it existed? | NA | Cross-sectional survey |
| 8. For exposures that can vary in amount or level, did the study examine different levels of the exposure as related to the outcome (e.g., categories of exposure, or exposure measured as continuous variable)? | Yes | Questionnaire comprised of 5-point Likert scales |
| 9. Were the exposure measures (independent variables) clearly defined, valid, reliable, and implemented consistently across all study participants? | Cannot determine | Source of questions not reported |
| 10. Was the exposure(s) assessed more than once over time? | No | Cross-sectional survey |
| 11. Were the outcome measures (dependent variables) clearly defined, valid, reliable, and implemented consistently across all study participants? | No | Knowledge, attitudes and acceptability variables not well defined or described |
| 12. Were the outcome assessors blinded to the exposure status of participants? | No | Study design did not allow for blinding |
| 13. Was loss to follow-up after baseline 20% or less? | NA | Cross-sectional survey |
| 14. Were key potential confounding variables measured and adjusted statistically for their impact on the relationship between exposure(s) and outcome(s)? | No | Descriptive summaries only |
| Comments on key limitations | Cross-sectional survey where variables were not well defined in the methods. Descriptive summaries only. | |

| **Reference:** **Weldearegay et al 2019** | **Rating** | **Description** |
| --- | --- | --- |
| 1. Was the research question or objective in this paper clearly stated? | Yes | “Assesses the quality of KMC services in Ethiopia and the factors associated with its appropriate initiation among low birth weight neonates." |
| 2. Was the study population clearly specified and defined? | Yes | “A national cross-sectional survey of all public hospitals, health centers and private facilities (higher clinics and above) that provided maternal and newborn health services and reported attending births in the past 12 months.” |
| 3. Was the participation rate of eligible persons at least 50%? | Yes | “Of the eligible 4,385 facilities in all nine regions and two city administrations in Ethiopia, 3,804 facilities were assessed (including 293 hospitals, 3,459 health centers and 52 clinics).” |
| 4. Were all the subjects selected or recruited from the same or similar populations (including the same time period)? Were inclusion and exclusion criteria for being in the study prespecified and applied uniformly to all participants? | Yes | All health facilities that provided maternal and newborn health services and reported attending births were included in the 2016 Ethiopian Emergency Obstetrics and Newborn care (EmONC) assessment |
| 5. Was a sample size justification, power description, or variance and effect estimates provided? | Yes | National survey targeting all health facilities |
| 6. For the analyses in this paper, were the exposure(s) of interest measured prior to the outcome(s) being measured? | No | Cross-sectional survey |
| 7. Was the timeframe sufficient so that one could reasonably expect to see an association between exposure and outcome if it existed? | NA | Cross-sectional survey |
| 8. For exposures that can vary in amount or level, did the study examine different levels of the exposure as related to the outcome (e.g., categories of exposure, or exposure measured as continuous variable)? | Yes | With applicable variables in the WHO Service Availability and Readiness Assessment (SARA) tool |
| 9. Were the exposure measures (independent variables) clearly defined, valid, reliable, and implemented consistently across all study participants? | Yes | Indicators adapted from the WHO Service Availability and Readiness Assessment (SARA) tool |
| 10. Was the exposure(s) assessed more than once over time? | No | Cross-sectional survey |
| 11. Were the outcome measures (dependent variables) clearly defined, valid, reliable, and implemented consistently across all study participants? | Yes | Review of facility registries, survival status at discharge |
| 12. Were the outcome assessors blinded to the exposure status of participants? | No | Study design did not allow for blinding |
| 13. Was loss to follow-up after baseline 20% or less? | NA | Cross-sectional survey |
| 14. Were key potential confounding variables measured and adjusted statistically for their impact on the relationship between exposure(s) and outcome(s)? | Yes | Factors associated with appropriate KMC initiation was explored using multivariable logistic regression models |
| Comments on key limitations | Well designed cross-sectional survey conducted nationally across Ethiopia. Methodology and results clearly reported and analyses adjusted for potential confounders. | |

**CONTROLLED INTERVENTION STUDIES (NIH quality assessment tools)**

| **Reference** | **1** | **2** | **3** | **4** | **5** | **6** | **7** | **8** | **9** | **10** | **11** | **12** | **13** | **14** | **Rating** |
| --- | --- | --- | --- | --- | --- | --- | --- | --- | --- | --- | --- | --- | --- | --- | --- |
| Bergh et al 2008 | N | Y | N | N | N | Y | Y | Y | Y | NR | Y | N | Y | Y | Fair |
| Cattaneo et al 1998 | Y | NR | N | N | N | N | Y | Y | NR | NR | Y | N | Y | CD | Poor |
| Ibe et al 2004 | N | NA | NA | NA | NA | Y | Y | Y | NR | NR | Y | Y | Y | NA | Poor |
| Pattinson et al 2005 | Y | Y | N | N | N | Y | Y | Y | NR | NR | Y | N | Y | Y | Fair |

*Y – Yes, N – No, CD – Cannot determine/ cannot tell, NR – not reported, NA – not applicable*

| **Reference:** **Bergh et al 2008** | **Rating** | **Description** |
| --- | --- | --- |
| 1. Was the study described as randomized, a randomized trial, a randomized clinical trial, or an RCT? | No | Not explicitly described by authors as a randomized trial |
| 2. Was the method of randomization adequate (i.e., use of randomly generated assignment)? | Yes | Randomization was achieved by spinning a coin |
| 3. Was the treatment allocation concealed (so that assignments could not be predicted)? | No | Treatment allocation concealment not possible with implementation intervention |
| 4. Were study participants and providers blinded to treatment group assignment? | No | Blinding not possible with implementation intervention |
| 5. Were the people assessing the outcomes blinded to the participants' group assignments? | No | Blinding not possible with implementation intervention |
| 6. Were the groups similar at baseline on important characteristics that could affect outcomes (e.g., demographics, risk factors, co-morbid conditions)? | Yes | Hospitals matched on characteristics before randomization “The hospitals were paired with  respect to their level of care, their geographical location (urban or rural) and the annual number of births at each facility (which varied between 200 and 7600 births per year). One hospital in each pair was randomly allocated to Group A, the other to Group B, by spinning a coin. Group A received on-site facilitation and Group B off-site facilitation.” |
| 7. Was the overall drop-out rate from the study at endpoint 20% or lower of the number allocated to treatment? | Yes | No drop-out of hospitals |
| 8. Was the differential drop-out rate (between treatment groups) at endpoint 15 percentage points or lower? | Yes | No drop-out of hospitals |
| 9. Was there high adherence to the intervention protocols for each treatment group? | Yes | The same two resource persons conducted the introductory workshop and attended almost all of the facilitation sessions, content followed an evidence-based workbook |
| 10. Were other interventions avoided or similar in the groups (e.g., similar background treatments)? | Not reported | Not reported if there were any other interventions |
| 11. Were outcomes assessed using valid and reliable measures, implemented consistently across all study participants? | Yes | Used a previously validated six-step model with progress markers for evaluation |
| 12. Did the authors report that the sample size was sufficiently large to be able to detect a difference in the main outcome between groups with at least 80% power? | No | Sample size calculations not discussed |
| 13. Were outcomes reported or subgroups analyzed prespecified (i.e., identified before analyses were conducted)? | Yes | Outcomes reported in methods. No sub-group analyses planned or reported in results |
| 14. Were all randomized participants analyzed in the group to which they were originally assigned, i.e., did they use an intention-to-treat analysis? | Yes | On-site and off-site groups were separate throughout the study |
| Comments on key limitations | Hospitals were matched on baseline characteristics and then randomized. Evaluations occurred 6 to 8 months after launching the process which may be insufficient time for institutionalization of implementation. Unclear whether sample size of 36 hospitals to compare two strategies is sufficiently large to be able to detect a statistical difference in outcome. Blinding and treatment allocation concealment is not possible within the study design. | |

| **Reference:** **Cattaneo et al 1998** | **Rating** | **Description** |
| --- | --- | --- |
| 1. Was the study described as randomized, a randomized trial, a randomized clinical trial, or an RCT? | Yes | Described in the as a randomized controlled trial |
| 2. Was the method of randomization adequate (i.e., use of randomly generated assignment)? | Not reported | Randomization method not reported |
| 3. Was the treatment allocation concealed (so that assignments could not be predicted)? | No | Treatment allocation concealment not possible with KMC intervention compared to conventional methods of care |
| 4. Were study participants and providers blinded to treatment group assignment? | No | Blinding not possible |
| 5. Were the people assessing the outcomes blinded to the participants' group assignments? | No | Blinding not possible |
| 6. Were the groups similar at baseline on important characteristics that could affect outcomes (e.g., demographics, risk factors, co-morbid conditions)? | No | A higher proportion of infants with gestational age <32 weeks in the KMC group in Addis Ababa |
| 7. Was the overall drop-out rate from the study at endpoint 20% or lower of the number allocated to treatment? | Yes | No loss to follow up after enrollment |
| 8. Was the differential drop-out rate (between treatment groups) at endpoint 15 percentage points or lower? | Yes | No loss to follow up after enrollment |
| 9. Was there high adherence to the intervention protocols for each treatment group? | Not reported | Compliance and adherence not reported |
| 10. Were other interventions avoided or similar in the groups (e.g., similar background treatments)? | Not reported | Not reported if there were any other interventions |
| 11. Were outcomes assessed using valid and reliable measures, implemented consistently across all study participants? | Yes | Described in the methods citing WHO definitions where applicable |
| 12. Did the authors report that the sample size was sufficiently large to be able to detect a difference in the main outcome between groups with at least 80% power? | No | Sample size calculations not discussed |
| 13. Were outcomes reported or subgroups analyzed prespecified (i.e., identified before analyses were conducted)? | Yes | Analysis plan and stratifying by site, sex, birthweight and socioeconomic variables described in methods |
| 14. Were all randomized participants analyzed in the group to which they were originally assigned, i.e., did they use an intention-to-treat analysis? | Cannot determine | 9 KMC infants at Addis Ababa transferred to conventional methods of care and “all returned to KMC as soon as the problem was solved” |
| Comments on key limitations | Only 45% of eligible infants in Addis Ababa was enrolled indicating potential sampling bias. Many in Addis were excluded because their mothers had abandoned them. Analyses not adjusted for potential confounders. Blinding and treatment allocation concealment is not possible within the study design. | |

| **Reference:** **Ibe et al 2004** | **Rating** | **Description** |
| --- | --- | --- |
| 1. Was the study described as randomized, a randomized trial, a randomized clinical trial, or an RCT? | No | An experimental study with a crossover design |
| 2. Was the method of randomization adequate (i.e., use of randomly generated assignment)? | NA | Not randomized trial |
| 3. Was the treatment allocation concealed (so that assignments could not be predicted)? | NA | Not randomized trial |
| 4. Were study participants and providers blinded to treatment group assignment? | NA | Not randomized trial |
| 5. Were the people assessing the outcomes blinded to the participants' group assignments? | NA | Not randomized trial |
| 6. Were the groups similar at baseline on important characteristics that could affect outcomes (e.g., demographics, risk factors, co-morbid conditions)? | Yes | Cross-over trial |
| 7. Was the overall drop-out rate from the study at endpoint 20% or lower of the number allocated to treatment? | Yes | No drop-out reported (13 infants recruited, results reported for all 13) |
| 8. Was the differential drop-out rate (between treatment groups) at endpoint 15 percentage points or lower? | Yes | No drop-out reported (13 infants recruited, results reported for all 13) |
| 9. Was there high adherence to the intervention protocols for each treatment group? | Not reported | Compliance and adherence to treatments not reported |
| 10. Were other interventions avoided or similar in the groups (e.g., similar background treatments)? | Not reported | Not reported if there were any other interventions |
| 11. Were outcomes assessed using valid and reliable measures, implemented consistently across all study participants? | Yes | Continuous ambulatory monitoring of the infant’s temperature over a 24-hour period was conducted using a compact, battery-powered Eltek 1000 series Squirrel Memory Data Logger. |
| 12. Did the authors report that the sample size was sufficiently large to be able to detect a difference in the main outcome between groups with at least 80% power? | Yes | Sample size calculations found that at least 20 paired measurements will be required to show a difference of at least 0.5 C between KMC and CC. Forty-eight (one in six) paired measurements were analysed for each infant |
| 13. Were outcomes reported or subgroups analyzed prespecified (i.e., identified before analyses were conducted)? | Yes | Outcomes reported in methods. No sub-group analyses planned or reported in results |
| 14. Were all randomized participants analyzed in the group to which they were originally assigned, i.e., did they use an intention-to-treat analysis? | NA | Cross-over trial |
| Comments on key limitations | Because this was a cross-over trial, there was no randomization or blinding in the study. Compliance and adherence to treatments was not reported. | |

| **Reference:** **Pattinson et al 2005** | **Rating** | **Description** |
| --- | --- | --- |
| 1. Was the study described as randomized, a randomized trial, a randomized clinical trial, or an RCT? | Yes | Described in the title as a randomized trial |
| 2. Was the method of randomization adequate (i.e., use of randomly generated assignment)? | Yes | Randomization was achieved by spinning a coin |
| 3. Was the treatment allocation concealed (so that assignments could not be predicted)? | No | Treatment allocation concealment not possible with implementation intervention |
| 4. Were study participants and providers blinded to treatment group assignment? | No | Blinding not possible with implementation intervention |
| 5. Were the people assessing the outcomes blinded to the participants' group assignments? | No | Blinding not possible with implementation intervention |
| 6. Were the groups similar at baseline on important characteristics that could affect outcomes (e.g., demographics, risk factors, co-morbid conditions)? | Yes | Hospitals matched on characteristics before randomization “hospitals were paired with respect to their geographical location (urban or rural) and annual number of births at the facility (varied between 300 and 10 000 births per year). One hospital in each pair was randomly allocated to either group A or B and the other received the alternate option” |
| 7. Was the overall drop-out rate from the study at endpoint 20% or lower of the number allocated to treatment? | Yes | No drop-out of hospitals |
| 8. Was the differential drop-out rate (between treatment groups) at endpoint 15 percentage points or lower? | Yes | No drop-out of hospitals |
| 9. Was there high adherence to the intervention protocols for each treatment group? | Not reported | Compliance and adherence to outreach strategies not reported |
| 10. Were other interventions avoided or similar in the groups (e.g., similar background treatments)? | Not reported | Not reported if there were any other interventions |
| 11. Were outcomes assessed using valid and reliable measures, implemented consistently across all study participants? | Yes | Used a previously validated six-step model with progress markers for evaluation |
| 12. Did the authors report that the sample size was sufficiently large to be able to detect a difference in the main outcome between groups with at least 80% power? | No | Sample size calculations not discussed |
| 13. Were outcomes reported or subgroups analyzed prespecified (i.e., identified before analyses were conducted)? | Yes | Outcomes reported in methods. No sub-group analyses planned or reported in results |
| 14. Were all randomized participants analyzed in the group to which they were originally assigned, i.e., did they use an intention-to-treat analysis? | Yes | Hospitals randomized to package without facilitation were offered facilitation after evaluation completion |
| Comments on key limitations | Hospitals were matched on baseline characteristics and then randomized. Evaluations occurred 8 months after launching the process which may be insufficient time for institutionalization of implementation. Unclear whether sample size of 34 hospitals to compare two strategies is sufficiently large to be able to detect a statistical difference in outcome. Blinding and treatment allocation concealment is not possible within the study design. | |
