# Supplementary material for: Barriers and facilitators of facility-based kangaroo mother care in sub-Saharan Africa: a systematic review
Source: BMC Pregnancy Childbirth. 2021 Mar 4;21:176. doi: 10.1186/s12884-021-03646-3 (PMC7934357; doi:10.1186/s12884-021-03646-3)
Supplement: Supplementary file 3 — Additional file 3. Barriers to KMC practice by study. [file 12884_2021_3646_MOESM3_ESM.docx]

**Additional file 3: Barriers to KMC practice by study**

| **Reference** | **Health system/ facility level** | **Health worker level** | **Family level** |
| --- | --- | --- | --- |
|  | **Lack of KMC guidelines or poor implementation**   - Absences of strong institutional policy, protocol or practice guidelines on KMC practice and roles - KMC thought to be preserve of NICU nurses because they are the ones who handle LBW/preterm babies | **Staffing shortages and workload**   - Workload on staff prevents them from helping mothers to practice KMC   **Inadequate knowledge on KMC and its benefits**   - More than half (39 out of 67, 58%) did not receive training - Perception that KMC is only for thermoregulation and bonding - Many nurses did not know that exclusive breastfeeding (35, 52%) and early discharge (46, 69%) were components of KMC - 24 nurses (36%) did not talk to mothers about KMC because they themselves do not have adequate knowledge | **Fears and discomforts**   - Mothers have low confidence because their babies are small - afraid their babies will fall due to their fragile small body size)   **Family attitudes and cultural beliefs**   - Mothers think practice is time-wasting - Mothers do not like KMC practice   **Stresses related to extended hospitalization**   - Long stay at the hospital and responsibilities at home - Some are petty traders and peasant farms who have to resume their trade in order to support their family |
| Aliganyira et al 2014 | **Lack of local leadership**   - Absence of internal support "So far I've only seen Save (the Children)" - Training, supportive supervision, resources provided by partners including Save the Children, UNICEF, Uganda Private Midwives Association, ISIS Foundation and Rotary International   **Unsupportive staffing policies**   - Staff attrition and rotation: only 22% of the staff reportedly trained in KMC within the last 3 years were still working with newborns at the time of the evaluation. - "This evaluation raises important questions about the investment in training health workers...Given Uganda's existing challenges with adequate human resources for health, this drain is costly and derails progress of institutionalisation"   **Lack of KMC guidelines or poor implementation**   - Only 3 hospitals could produce a written feeding policy, only one officially designed as baby-friendly hospital - Only two hospitals had discharge checklists or procedures   **Poor supportive supervision and record-keeping**   - Written evidence of expressed breastmilk feeding in only 3 facilities, | N/A | N/A |
| Bergh and Pattinson 2003 | **Lack of local leadership**   - KMC is not part of the institutional culture | **Staffing shortages and workload**   - Staff shortages   **Health worker attitudes and non-acceptance**   - The individual interested in KMC is not on duty | N/A |
| Bergh et al 2008 | **Lack of local leadership**   - Lack of sufficient opinion leaders who were convinced of the value of the program - Lost of leadership such as the departure of KMC implementation team leader one month after introductory workshop at one site that was not successful in implementing   **Inadequate facilities and supplies**   - Lack of dedicated KMC space   **Unsupportive staffing policies**   - When nursing staff is not rearranged to include supervision for KMC - Staff rotation policies and ability to orientate new staff | **Staffing shortages and workload**   - Low morale of health care staff where there were heavy workloads, staffing shortages, administrative constraints and high staff turnover | N/A |
| Bergh et al 2012 | **Lack of local leadership**   - Level of support and interest by management - Requires financial, material and human resources   **Inadequate facilities and supplies**   - Hospital setting space and equipment   **Preterm infants not prioritized**   - Requires educational opportunities for promoting KMC in health facilities, communities and districts | **Staffing shortages and workload**   - Poor collaboration and teamwork of staff - Staffing shortages   **Inadequate knowledge of KMC and its benefits**   - Gaps in training   **Health worker attitudes and non-acceptance**   - Attitudes | **Family attitudes and cultural beliefs**   - Lack of compliance and acceptance of mothers - Non-supportive cultural and religious beliefs |
| Bergh et al 2013 | **Poor supportive supervision and record-keeping**   - While a feeding job-aid for LBW infants were created and displayed, unclear if it was being used or followed   **Inadequate facilities and supplies**   - Environment not comfortable for mothers: few hospitals had low beds, provided pillows or comfortable chairs - Most facilities did not provide food for mothers)   **Lack of KMC guidelines or poor implementation**   - Lack of written checklist for procedures on admission to KMC and protocols on KMC practice - Few hospitals had consistent discharge criteria to help with effective discharge decisions   **Unsupportive staffing policies**   - Lack of long-term training plan to ensure all health workers were trained in KMC | **Inadequate knowledge of KMC and its benefits**   - On-the-job training was in variable quality and forms | **Family attitudes and cultural beliefs**   - Mothers insisted on going home and did not stay in the hospital for long after delivery - Cultural practices dictating that all infants be discharged from hospital within 24 hours   **Stresses related to extended hospitalization**   - Requirement of family support for mothers daily needs, associated with additional costs   **Poor support or negative interactions with medical staff**   - Mothers did not have access to adequate review policies, |
| Bergh et al 2014 | **Lack of local leadership**   - Deterioration in quality of services after end of project when scale up of KMC was linked to a donor project ("almost everything has faded")   **Lack of KMC guidelines or poor implementation**   - Lack of job-aids or protocols to guide management of KMC beyond admission and discharge criteria   **Poor supportive supervision and record-keeping**   - Absence of supportive supervision due to staff workload, lack of transport, distance between district office and health facilities and internal conflict between different health structures of authorities as a result of decentralization policies - Recommendations from review meetings not followed up - Lack of standardized reporting on KMC to higher levels - Poor quality of record keeping   **Preterm infants not prioritized**   - KMC is not included in antenatal care | **Inadequate knowledge of KMC and its benefits**   - Staff uncertainty about certain aspects of KMC practice due to insufficient depth of in-service orientation and lack of experience in caring for preterm infants in low-caseload facilities - Only 41% facilities reported orientation program in KMC for new staff - Variable quality of trainers reported with some trainers lacking knowledge, skills and experience, - Lack of clarity on what transpires during training | **Family attitudes and cultural beliefs**   - Low uptake of maternal and newborn services - Cultural beliefs such as baby should be carried on the back |
| Cattaneo et al 1998 | **Inadequate facilities and supplies**   - Inadequate supplies for KMC - Inadequate cooking and eating facilities for families during their stay at the hospital | N/A | **Stresses related to extended hospitalization**   - Complained about long stay in hospital - 29% mothers in Addis worried about care of their other children left a home |
| Chavula et al 2017 | **Inadequate facilities and supplies**   - Lack of defined space and functional weighing scale (especially at mission and community hospitals) - Consistently low supply of caps and hats for newborn (families expected to bring their own)   **Unsupportive staffing policies**   - Lack of staffing for KMC   **Lack of KMC guidelines or poor implementation**   - Lack of KMC guidelines   **Poor supportive supervision and record-keeping**   - Missing KMC registers (only central hospitals had up-to-date registers) | N/A | N/A |
| Chisenga, Chalanda, and Ngwale 2015 | **Unsupportive staffing policies**   - Lack of trained staff in the KMC unit as full time staff were either patient attendants or students - Nurse may come in to give support but would otherwise assigned to the NICU - No clinician support in the KMC unit | N/A | **Lack of awareness of KMC prior to hospitalization**   - Lack of previous awareness of KMC services before hospitalization in 84% of mothers in the study   **Stresses related to extended hospitalization**   - Long stay at the hospital: 43% participants had already spent one and half weeks in NICU waiting for infants to be stable before moving to KMC unit   **Lack of decision-making power for mothers**   - Husbands, mothers and mother-in-law had most influence on decision   **Fears and discomforts**   - Not comfortable with KMC position - Felt the infant was too small, especially with young/first time mothers - Younger the participant, the more uncomfortable she was with the KMC position - Lack of recreational activities leading to boredom   **Poor support or negative interactions with medical staff**   - Lack of assistance with skin-to-skin contact and breastfeeding,   **Family attitudes and cultural beliefs**   - preference of incubator care over KMC, |
| Davidge 2009 | **Inadequate facilities and supplies**   - Not much space between beds - No KMC jackets   **Lack of local leadership**   - Dependent on champions: "I am no longer in charge of the unit… Unfortunately, my old unit is now leaderless as they have not appointed anyone in my place. There is a huge turnover of staff and no one is really promoting or teaching KMC. It is still practised sporadically but is not a focus of care" | **Staffing shortages and workload**   - Busy and short-staffed unit   **Inadequate knowledge of KMC and its benefits**   - Poor trainer who didn't present the concept very positively - "I was horrified that any one in their right mind could propose that we remove sick babies from perfectly good incubators and put them on their mother's chest. These were sick infants that needed intensive care and monitoring -- how could this be done on a mother's chest? We ran a top class unit -- surely there was no place for KMC here? It seemed archaic and impractical with no apparent benefits..."   **Health worker attitudes and non-acceptance**   - Resented the extra work with a proscriptive implementation plan that emphasized monitoring with many forms to be filled, audit tools and an external inspection | **Family attitudes and cultural beliefs**   - Mothers traditionally carry their babies on their backs, not their front |
| Eddy and McInerney 2007 | N/A | N/A | **Fears and discomforts**   - Mothers described being afraid, anxious and confused before starting KMC - First time seeing it being done, most mothers doubted that it would work - Scared to handle their small babies |
| Feucht, et al 2015 | N/A | N/A | N/A |
| Gondwe et al 2016 | **Inadequate facilities and supplies**   - Lack of space to initiate KMC   **Unsupportive staffing policies**   - Although shortage of staff was widely acknowledged by the 5 policy makers interviewed, one (20 %) of them felt that duty allocation accelerated the problem - Concern that even knowledge gained through workshops was not well utilized in many health facilities because of how service providers were allocated in the hospital wards. As asserted by one service provider, ‘Sometimes there is one person selected to attend a training or workshop on KMC but you find that the person is no longer working in the postnatal ward to manage preterm infants. In this way, it does not help because the information is not well utilised’   **Poor supportive supervision and record-keeping**   - Logistics problems that limited policy makers to conduct timely supervision to health facilities   **Preterm infants not prioritized**   - Preterm birth was rarely mentioned during antenatal care - Little attention on the issue of managing preterm infants at district level resulting into less budgeting towards preterm infants   **Lack of KMC guidelines or poor implementation**   - Seven (63.6 %) of the service providers in health centres and district hospitals reported lack of knowledge of the existing policy protocol documents - None of them had a copy of the existing documents and they reported that the policy protocol documents were kept somewhere in other offices | **Staffing shortages and workload**   - Shortage of staff was widely acknowledged by the 5 policy makers interviewed   **Health worker attitudes and non-acceptance**   - An attitude problem among some of the service providers that a preterm infant would not survive. The service provider said, ‘As nurses, we also should accept that sometimes we have bad attitude …by just looking at the preterm baby and see how it looks, we say but is this one going to survive? Instead of either instructing the mother on what to do or checking on the baby you just stay, that the mother will come and ask, we sometimes have a problem as well” - There was an attitude problem among some of the health workers as they were not willing to learn from colleagues and utilise the available policy protocol documents to guide them on appropriate care. For instance, policy makers described providing enough support, including trainings and materials to health workers on care for preterm infants, but when monitoring them, care was not conducted as expected | **Stresses related to extended hospitalization**   - Additional costs to families for hospital stay: Some women were reported too poor to afford linen to cover their preterm infants   **Family attitudes and cultural beliefs**   - most mothers would not follow instructions from service providers but rather listen to their guardians on caring for their preterm infants |
| Ibe et al 2004 | N/A | N/A | **Family attitudes and cultural beliefs**   - Nursing a tiny infant in skin-to-skin contact between the mother’s breasts is an uncommon practice in Nigeria - Local belief is that tiny infants are better swaddled and nursed in warm rooms, with minimal contact with their mothers   **Fears and discomforts**   - 5 out of 8 (63%) reported problems with KMC |
| Kambarami, Chidede and Kowo 1999 | **Inadequate facilities and supplies**   - Shortage of KMC beds, shortage of trained nursing staff | **Staffing shortages and workload**   - Shortage of trained nursing staff   **Inadequate knowledge of KMC and its benefits**   - Lack of confidence in referring eligible infants due to new innovation ("The mean age at admission to the KCU was 12 days. This major delay in referring well preterm infants to the KCU was mainly because of over caution given that this method of care was newly introduced in this hospital")   **Health worker attitudes and non-acceptance**   - Poor morale among nursing staff and doctors | **Fears and discomforts**   - Maternal anxiety when nurses were not confident |
| Kambarami, Mutambirwa and Maramba 2002 | N/A | N/A | **Fears and discomforts**   - Mothers said it was difficult to sleep with an infant on their chest at first   **Poor support or negative interactions with medical staff**   - Feeding was a challenge as infants sometimes aspirate when they used cups   **Stresses related to extended hospitalization**   - Lack of food as hospital did not feed mothers and husbands could not always get time off from work to bring food (mothers felt the lack of food affected their milk supply)   **Lack of decision-making power for mothers**   - Some husbands discouraged the practice because felt the infant was too fragile or not keen on the change of method of care ("preterm infants have always been there so wat's new, why change the method of care?") - Grandmothers were very skeptical because they had never heard of it before and some thought that mothers were making excuses for not doing the routine household chores, |
| Kampekete, Ngoma and Masumo 2018 | **Inadequate facilities and supplies**   - Lack of KMC wrappers | **Staffing shortages and workload**   - Staffing shortages hindered ability to train staff "as there would be no one to supervise mothers"   **Inadequate knowledge of KMC and its benefits**   - Lack of training in health workers overseeing the management of the KMC unit | **Stresses related to extended hospitalization**   - Irregular financial or material support from family members   **Poor support or negative interactions with medical staff**   - 90% were practicing continuous KMC but only 65% said the information they received was adequate |
| Kiwanuka et al 2017 | **Inadequate facilities and supplies**   - Noisy and overcrowded with no incubators for mothers to keep their babies when they want to rest or take a bath - Beds in the ward had no pillows and could not be adjusted for sleeping - No side chairs for mothers to breastfeed | N/A | **Poor support or negative interactions with medical staff**   - Families reported little assistance from the health care workers including on how to hold their infants in KMC position   **Lack of awareness of KMC prior to hospitalization**   - Two-thirds of mothers had never heard about KMC before giving birth to premature babies and begin transferred to the neonatal ward   **Fears and discomforts**   - All mothers shared that they became fatigued practicing KMC because of poor sleep, lack of assistance from relatives and long hours in the KMC position |
| Leonard and Mayers 2008 | N/A | N/A | **Lack of awareness of KMC prior to hospitalization**   - Unexpectation of preterm birth - "Parents feel cheated out of a full-term 'normal' birth. The labour occurs unexpectedly" - Usually little if any preparation for preterm birth and parents unprepared - Lack of knowledge about KMC before delivery   **Fears and discomforts**   - Parents felt incompetent wen initially entering the NICU as an unfamiliar and intimidating environment with complex medical equipment and constant activity - Feared hurting fragile infant - Felt clumsy and anxious when they first held their infant in KMC position - Fathers felt judged and criticised in their efforts to provide KMC and initially felt useless and "not really part of it", - Feeling hopeless when results are not visible: "Successful care of a stable preterm infant is determined primarily by weight gain. Parents perceive weight gain as a measure of success, progress and development.... Failure of gain weight when the measure of success is weight gain impacts on the mother mentally and emotionally. All she can do is hold and feed her infant. There is a sense of helplessness; they can only do so much and they want to do more." - "Backache, boredom, loneliness, tiredness and anxiety become an integral part of the living-in experience." - Isolation of mother from family support while staying at the hospital |
| Lincetto, Nazir and Cattaneo 2000 | N/A | **Staffing shortages and workload**   - "Nurses complained that they had no time to check temperature, to weigh infants, to supervise breastfeeding and to talk with mothers, especially in the afternoon and night shifts when two nurses had to deal with 60-80 patients" - KMC increased workload for nurses through new routines and improved survival and increased referral from peripheral maternity units   **Inadequate knowledge of KMC and its benefits**   - Staff unfamiliar with KMC implementation   **Health worker attitudes and non-acceptance**   - Staff skepticism: "believed that only between equipment of supplies would improve survival of LBWI" | **Family attitudes and cultural beliefs**   - Low birth weight infants were not accepted by mothers, seen as 'ugly' or 'not normal' - Feeding tube 'hurt the baby' - Colostrum considered 'dirty' milk |
| Morgan et al 2018 | **Inadequate facilities and supplies**   - Lack of monitoring devices - Lack of beds and space in newborn unit | **Staffing shortages and workload**   - Monitoring is a challenge due to staffing shortages | **Family attitudes and cultural beliefs**   - Stigma and guilt related to having a premature infant - Lack of motivation among mothers to devote time   **Poor support or negative interactions with medical staff**   - Lack of KMC education   **Stresses related to extended hospitalization**   - Lack of family support and lack of money for mothers to buy food - Difficulties with KMC related to cost and increased time away from work for father   **Fears and discomforts**   - Concerns about pain and dislodging tubing during KMC   **Maternal medical conditions**   - Difficulties of KMC following a caesarean delivery |
| Namazzi et al 2016 | N/A | **Inadequate knowledge of KMC and its benefits**   - Training difficult when high turnover amongst staff | **Lack of awareness of KMC prior to hospitalization**   - Lack of exposure to KMC as a treatment regimen   **Stresses related to extended hospitalization**   - Additional cost to families (e.g. meals) for inpatient care - Care for children at home |
| Onubogu and Okoh 2016 | **Lack of KMC policies, guidelines and job aids**   - Lack of policy on the practice of KMC - Most common reason for not practicing KMC, reported by 58.9% of 73 respondents who did not practice   **Inadequate facilities and supplies**   - Not having suitable environment to practice KMC: 41.1%, second leading reason - Lack of KMC wrappers: 20.5%, third leading reason | **Inadequate knowledge of KMC and its benefits**   - Lack of training was described   **Health worker attitudes and non-acceptance**   - Training was described in only 12.3% of respondents who did not practice so motivation to practice within a supportive environment may important factors | **Stresses related to extended hospitalization**   - Transferred cost of KMC ward occupancy on the family who already has the financial burden of a long stay preterm infant |
| Pattinson et al 2005 | N/A | N/A | N/A |
| Söderbäck and Erlandsson 2012 | **Poor supportive supervision and record-keeping**   - Provisioning of training on KMC to mothers depended if the nurse felt like it (nurse's own willingness, sympathy and kindliness on admission) suggesting a lack of follow-up by supervisors | **Health worker attitudes and non-acceptance**   - Attitude of the nurse on shift influenced training mothers on KMC (provisioning depended on nurse's own willingness, sympathy and kindliness on admission) | **Poor support or negative interactions with medical staff**   - Culture of not passing on information and disempowering mothers - Nurses communication towards the mothers was brief and postulating - Mother reported that doctors were difficult to understand - Lack of understanding of KMC while in the nursery ward,   **Lack of awareness of KMC prior to hospitalization**   - None of the mothers reported receiving any information before transfer to the nursery ward about what KMC involved |
| Solomons and Rosant 2012 | **Inadequate facilities and supplies**   - Insufficient space   **Lack of KMC guidelines or poor implementation**   - A majority of nursing staff knew a policy existed but not whether their facilities complied with the policy | **Inadequate knowledge of KMC and its benefits**   - Lack of KMC training of all nursing staff (antenatal clinic and hospital) | **Lack of awareness of KMC prior to hospitalization**   - Not receiving any information regarding KMC at ANC - Mothers of premature infants often completely unprepared to cope with their newborn infants   **Stresses related to extended hospitalization**   - Resistance due to being separated from their families for long periods of time - Concerns about the care of their other children - Difficult for mothers of twins without companion support and engaging family members   **Fears and discomforts**   - Isolation: not receiving any visitors - Difficulty sleeping with infants on chest |
| ten Ham, Minnie and van der Walt 2016 | **Lack of local leadership**   - Lack of leadership on provincial level - Lack of leadership by facility management | N/A | N/A |
| Watkins et al 2018 | **Inadequate facilities and supplies**   - Lack of suitable environment was the reason for intermittent SSC rather than continuous 75% of the time - Overcrowding, lack of privacy or shortage of chairs for families, lack of privacy - There was a small KMC room was available but not used | N/A | **Lack of awareness of KMC prior to hospitalization**   - Lack of previous awareness of KMC services before hospitalization   **Maternal medical conditions**   - Mother was ill |
| Weldearegay et al 2019 | N/A | **Staffing shortages and workload**   - Staffing shortages | N/A |
