# Supplementary material for: Barriers and facilitators of facility-based kangaroo mother care in sub-Saharan Africa: a systematic review
Source: BMC Pregnancy Childbirth. 2021 Mar 4;21:176. doi: 10.1186/s12884-021-03646-3 (PMC7934357; doi:10.1186/s12884-021-03646-3)
Supplement: Supplementary file 4 — Additional file 4. Facilitators of KMC practice by study. [file 12884_2021_3646_MOESM4_ESM.docx]

**Additional file 4: Facilitators of KMC practice by study**

| **Reference** | **Health system/ facility level** | **Health worker level** | **Family level** |
| --- | --- | --- | --- |
| Adzitey et al 2017 | **KMC policies and protocols**   - Strong policies to support KMC implementation: a well-documented protocol on KMC practice, KMC should be part of NICU protocols, written practice guidelines),   **Supportive staffing policies**   - Appointment of KMC nurses   **Sufficient space and supplies**   - NICU nurses suggested that KMC rooms should be made conductive for KMC practice (reclining chairs and beds) | **Adequate training and understanding of KMC benefits**   - All nurses thought that KMC training is essential - Almost all nurses (62, 93%) agreed that HIV positive mothers can safely provide KMC | **Family support and visits**   - Other family members can be asked to provide KMC when mother is sick or otherwise unable to, educating spouse and family members with mothers in KMC education |
| Aliganyira et al 2014 | **Local leadership support**   - Involvement of senior management: hospital directors, medical superintendents, head nurses and nursing managers - Involvement of senior management to support allowing staff to be trained, material support such as space alterations and showing personal interest - "He (director) was there for us - support and was in all meetings"   **Supportive supervision and dedicated registers**   - Evidence of record keeping in all health facilities including locally-adapted KMC registers | N/A | N/A |
| Bergh and Pattinson 2003 | N/A | **Staff acceptability and enthusiasm**   - Requires “a major paradigm shift from key role players and management. Healthcare workers are no longer in “control”, as the primary care of the infant is given back to the mother, who is provided with a supportive environment by the health professionals. It is not “our” baby any more, but the mother’s baby.” | N/A |
| Bergh et al 2008 | **Local leadership support**   - Strong management support: CEO of hospital signed undertaking to implement the program - Multidisciplinary team of health workers who acted as resource persons responsible for facilitation at, interaction with and feedback to all hospitals - Face-to-face facilitation which is not crucial if it takes place at a centre of excellence or at the hospital where the new programme was implemented if there was strong management support | N/A | N/A |
| Bergh et al 2012 | **Local leadership support**   - Use of steering committees to drive and supervise the implementation process, formalized in regional KMC steering committees that included a representative from each district - Tools and materials developed during workshops were collated with all materials owned by the workshop participants and immediately accessible for use - Use of cell phone messages to encourage steering committee members in their KMC implementation work - Multidisciplinary teams | **Staff acceptability and enthusiasm**   - Enthusiastic staff in some areas - Collaboration and teamwork | **Family support and visits**   - Good support for mothers with LBW infants |
| Bergh et al 2013 | **Local leadership support**   - Support from management - Efficient functioning of the regional KMC steering committee   **KMC policies and protocol**   - Written documents and guidelines for strengthening KMC   **Supportive staffing policies**   - Not rotating staff   **Supportive supervision and dedicated registers**   - Improved communication between KMC midwives and maternity and hospital management - Improvement of record keeping and statistics through development of a special register or collective record for infants receiving KMC - Was associated with the hospitals that were able to provide figures for the number of infants who received KMC   **Integration into maternal health services**   - Better integration of KMC into antenatal activities, | N/A | **Knowledge of KMC benefits**   - Concept and practice generally acceptable to mothers who were able to cite benefits of KMC - Success stories of LBW infants who survived having been nursed in KMC was motivational |
| Bergh et al 2014 | N/A | **Adequate training and understanding of KMC benefits**   - Pre-service curricula of nursing, medical and other clinical staff complemented by in-service training | **Family support and visits**   - Promotion of companions in the care of mother and baby |
| Cattaneo et al 1998 | **Sufficient space and supplies**   - Dedicated space for KMC   **Low cost**   - Cost-effectiveness of KMC compared to conventional care: running costs was about 50% less for KMC than conventional care (especially for electricity to keep warm room in Addis Ababa) | **Staff acceptability and enthusiasm**   - High staff acceptability: 100% of staff in Addis Ababa chose KMC over conventional methods of care | **Knowledge of KMC benefits**   - Bonding and appreciated being able to spend time with their babies   **Family support and visits**   - Daily/ frequent visits from family members partially relieved worries of home |
| Chavula et al 2017 | **Supportive supervision and dedicated registers**   - Register and monthly report form to track KMC services has been developed | N/A | N/A |
| Chisenga, Chalanda, and Ngwale 2015 | N/A | N/A | **Knowledge of KMC benefits**   - Awareness and counselling prior to KMC practice to increase family knowledge of the practice helped to increase acceptance - Counselling was done with 81% of participants prior to utilization and mothers interviewed were able to identify activities that take place in facility-based KMC (weighing infants, skin-to-skin care, exclusive breastfeeding and cleaning infants) - Agreed that KMC was beneficial to infant (protect infant, KMC saves lives, good for health, for fast growth, because nurse had said so) |
| Davidge 2009 | **KMC policies and protocol**   - Provincial guidelines developed,   **Sufficient space and supplies**   - Comfortable space for KMC (day room with TV, dining area, bedlinen, folding chairs, minimum spacing requirements) - Distributing simple KMC jackets   **Supportive staffing policies**   - Day to day care is provided by nursing axillaries with a nurse available for consultation or the administration of medications. | **Adequate training and understanding of KMC benefits**   - Health worker understanding of the benefits - "Finally, I understood the objectives - decreased infection, more successful breastfeeding, improved homeostasis and decreased hospital stays. These were things for which I could advocate. It made sense" | N/A |
| Feucht, et al 2015 | **Local leadership support**   - Focused intervention to strengthen KMC services through multidisciplinary district clinical specialist teams   **Sufficient space and supplies**   - Conducive KMC environment with appropriate time and space to provide services - Provisioning KMC wraps   **KMC policies and protocol**   - Onsite protocols and guidelines   **Supportive supervision and dedicated registers**   - Continuous supportive supervision by the district clinical specialist teams   **Integration into maternal health services**   - Integration of KMC with other maternal and child health programs at facility level (i.e. family planning, HIV prevention and immunisation services) | **Mentorship and opportunities to share knowledge**   - Empowerment of staff through workshops and mentorship visits through non-hierarchical approach | **Sense of empowerment and increased confidence**   - Empowering mothers to take responsibility for the care of their LBW and preterm babies under medical and nursing supervision   **Received support from medical staff**   - 'Special-needs groups' with social or medical risk factors |
| Gondwe et al 2016 | **KMC policies and protocol**   - All (5) policy makers reported that there was SRHR policy protocol document and quoted its policy statement that ‘Kangaroo mother care shall be routinely used in the management of premature infants’ - Copies of the SRHR policy were distributed to all health facilities in Malawi | **Adequate training and understanding of KMC benefits**   - Health workers use knowledge gained through their nursing training period, meetings, and workshops | N/A |
| Ibe et al 2004 | N/A | N/A | **Knowledge of KMC benefits**   - Mothers said KMC was safe, convenient for mother, comfortable for mother and baby, does not feel separated from baby - Happy to be able to start breastfeeding earlier |
| Kambarami, Chidede and Kowo 1999 | N/A | **Adequate training and understanding of KMC benefits**   - Training was associated with more confidence in assessing preterm infants for eligibility and less delays in referral to the KMC unit, which was associated with better outcomes. | **Received support from medical staff**   - Emotional support and constant recurrence from nursing staff that the infants were doing well |
| Kambarami, Mutambirwa and Maramba 2002 | N/A | N/A | **Knowledge of KMC benefits**   - Mother's willingness to follow nurses instructions and trust that it will help their baby grow, shield from infections and allowed infants to be fed on demand - Believed that baby was comfortable on mother's chest: slept longer on the chest and tended to cry more when put down - Shorter hospital stay than when baby was put on incubator which mothers said helped to keep down hospital costs   **Sense of empowerment and increased confidence**   - "most mothers said they felt that KMC made them feel that they were contributing more to the care of their infant than when infants were nursed in incubators”   **Family support and visits**   - Most mothers said their husbands were very supportive of the kangaroo care method |
| Kampekete, Ngoma and Masumo 2018 | **Sufficient space and supplies**   - Increased KMC bed space | **Adequate training and understanding of KMC benefits**   - More trained staff to offer continuous information and education to mothers on KMC - Incorporating KMC into pre-service curricula for health professionals | **Knowledge of KMC benefits**   - High knowledge of KMC - Mothers with high knowledge of KMC were 288% times more likely to accept the intervention, (OR 3.875, p=0.02) and did not think it was wrong to put the baby naked-skin to skin   **Family support and visits**   - Receiving financial or material support from family members - Supportive partners/fathers particularly in stressful post-birth period when mothers fear losing their infants - Approval by mother/mother-in-law and/or husband of the practice - 90% of fathers agreed with mothers practicing KMC |
| Kiwanuka et al 2017 | N/A | N/A | **Peer support from other mothers**   - Instruction from other mothers on how to position baby in KMC |
| Leonard and Mayers 2008 | N/A | N/A | **Sense of empowerment and increased confidence**   - Family confidence increased with KMC practice and time spent with their infant - Bonding with infant: "parents comfort and transfer strength, courage and hope to their infant through their touch…there is an intimacy between the parent and infant"   **Received support from medical staff**   - Maternity ward staff becoming companions to the parents and sharing their journey with them   **Peer support from other mothers**   - Support from other mothers |
| Lincetto, Nazir and Cattaneo 2000 | **Local leadership support**   - Support of hospital and provincial health authorities - Managerial support in supervision and solving problems   **Supportive staffing policies**   - Additional support by pediatrician: second rounds in the afternoon and being on call until 10pm for neonatal emergencies and sometimes performing nurses' tasks   **Supportive supervision and dedicated registers**   - "Progressive involvement of doctors and nurses in the care of LBWI with KMC, careful collection of data, along with periodic discussion of results, eventually succeeded in overcoming this resistance" | **Adequate training and understanding of KMC benefits**   - continuous on-the-job training   **Mentorship and opportunities to share knowledge**   - "Progressive involvement of doctors and nurses in the care of LBWI with KMC, careful collection of data, along with periodic discussion of results, eventually succeeded in overcoming this resistance"   **Staff acceptability and enthusiasm**   - Feeling proud that they were able to successfully deal with LBWI - Saw increased referral s as sign of recognition of their new skills | **Receiving support from medical staff**   - Talking to mothers during weight monitoring in the hospital and follow-up visits after discharge to understand their fears and beliefs, help them accept the baby and trust in survival, justify the requirements of KMC and help find solutions to their problems   **Peer support from other mothers**   - Experienced mothers as advocates for newly admitted mothers |
| Morgan et al 2018 | **Sufficient space and supplies**   - Availability of monitoring devices - Provisioning of meals in the newborn unit   **Integration into maternal health services**   - Community outreach and education about KMC in antenatal clinics | **Adequate training and understanding of KMC benefits**   - Health providers had general knowledge about KMC and its benefits - Cited benefits as to provide warmth to preterm babies - Said that it promotes breastfeeding and infant bonding - Felt that KMC improved monitoring of unstable neonates | **Knowledge of KMC benefits**   - Parents had general knowledge about KMC and its benefits - Cited benefits as providing warmth to preterm babies - Agreed that it promotes breastfeeding and infant bonding - Felt that KMC led to improved infant monitoring   **Sense of empowerment and increased confidence**   - Practicing KMC gave them a sense of responsibility in caring for their babies   **Receiving support from medical staff**   - Staff counselling   **Peer support from other mothers**   - Peer counselling   **Family support and visits**   - Family support |
| Namazzi et al 2016 | **Sufficient space and supplies**   - Dedicated space for KMC | **Mentorship and opportunities to share knowledge**   - Development of champions to support newly introduced KMC services (short-term solution) | N/A |
| Onubogu and Okoh 2016 | **KMC policies and protocol**   - A clear written protocol to standardize the decision of who qualifies for the care, where it should be carried out and discharge procedure | N/A | **Lowering hospital costs to families**   - KMC wards should be subsidized and charged at biweekly and monthly rates - Mothers who stay longer should be given higher discounts |
| Pattinson et al 2005 | **Local leadership support**   - Support, administrative commitment and endorsement by important opinion leaders | **Adequate training and understanding of KMC benefits**   - Face-to-face facilitation delivered with a multimedia implementation package | N/A |
| Reddy and McInerney 2007 | N/A | N/A | **Knowledge of KMC benefits**   - Receiving information through talks and demonstrations - Use of charts and pictures of KMC enhanced family understanding   **Sense of empowerment and increased confidence**   - Weight gain triggered feelings of elation and excitement while practicing KMC - Feelings of determination and commitment by mothers   **Received support from medical staff**   - Help from staff to attach their bodies to their chests   **Peer support from other mothers**   - Receiving encouragement from other mothers   **Family support and visits**   - Support from family |
| Söderbäck and Erlandsson 2012 | **Sufficient space and supplies**   - Each mother had their own bed to share with her baby/babies | N/A | N/A |
| Solomons and Rosant 2012 | **Local leadership support**   - Commitment from managers and their personnel   **Sufficient facilities and supplies**   - More facilities such as lockets and cupboards, a laundry area and dining area, proper beds to increase mothers' comfort | **Adequate training and understanding of KMC benefits**   - Understood benefits of KMC practice - In particular, reported that it promoted mother-infant bonding, enhanced the mother's confidence with regards to how to handle her LBW infant, and resulted in effective breastfeeding   **Staff acceptability and enthusiasm**   - Accepted because of benefits and did not think that KMC would increase their workload | **Knowledge of KMC benefits**   - Mothers feel positive towards KMC and found the practice acceptable   **Peer support from other mothers**   - Reminding each other about the importance of KMC - Discussed how to comfort their babies and how to kangaroo the infants properly - Exchanged ideas on how to minimize discomfort)   **Receiving support from medical staff**   - Continuous support and education from nurses |
| ten Ham, Minnie and van der Walt 2016 | **Local leadership support**   - Senior management buy-in, such as by the provincial head - Government (i.e. National Department of Health) buy-in - Hospital management, provincial and national support for human resources infrastructure, budget, equipment, policies and guidelines   **KMC policies and protocol**   - Hospital protocols aligned with provincial protocols and national policies   **Supportive supervision and dedicated registers**   - Benchmarking visits by management | **Adequate training and understanding of KMC benefits**   - Experienced KMC facilitators with clinical experience to conduct trainings - KMC included in pre-service curricula   **Staff acceptability and enthusiasm**   - Nursing staff buy-in   **Mentorship and opportunities to share knowledge**   - Sharing knowledge and training through training, conferences, networking, posters, surveys and workshops - National awareness campaigns and annual congresses to communicate best practices | **Family support and visits**   - Patient and family buy-in |
| Watkins et al 2018 | N/A | N/A | **Family support and visits**   - Grandmothers and aunts played an important role on the day of birth when mother was recovering from labour |
| Weldearegay et al 2019 | **Sufficient facilities and supplies**   - Facilities with separate newborn corners were more likely to initiate KMC services compared to their counterpart (AOR = 1.49, 95% CI: 1.06–2.10).     **Supportive staffing policies**   - Facilities that had a policy in place for staff rotation in newborn care (more than one rotation a year) were 1.84 times more likely to initiate KMC compared to facilities without staff rotation policies (AOR = 1.84, 95% CI: 1.34–2.51) | N/A | N/A |
